# A Multicassette Gateway Vector Set for High Throughput and Comparative Analyses in *Ciona* and Vertebrate Embryos

**DOI:** 10.1371/journal.pone.0000916

**Published:** 2007-09-19

**Authors:** Agnès Roure, Ute Rothbächer, François Robin, Eva Kalmar, Giustina Ferone, Clément Lamy, Caterina Missero, Ferenc Mueller, Patrick Lemaire

**Affiliations:** 1 Institut de Biologie du Développement de Marseille Luminy, UMR 6216 CNRS/Université de la Méditerranée, Marseille, France; 2 Institute of Toxicology and Genetics, Forschungszentrum Karlsruhe, Karlsruhe, Germany; 3 CEINGE Biotecnologie Avanzate SCarl (Center for Genetic Engineering), Napoli, Italy; Ecole Normale Supérieure de Lyon, France

## Abstract

**Background:**

The past few years have seen a vast increase in the amount of genomic data available for a growing number of taxa, including sets of full length cDNA clones and cis-regulatory sequences. Large scale cross-species comparisons of protein function and cis-regulatory sequences may help to understand the emergence of specific traits during evolution.

**Principal Findings:**

To facilitate such comparisons, we developed a Gateway compatible vector set, which can be used to systematically dissect cis-regulatory sequences, and overexpress wild type or tagged proteins in a variety of chordate systems. It was developed and first characterised in the embryos of the ascidian *Ciona intestinalis*, in which large scale analyses are easier to perform than in vertebrates, owing to the very efficient embryo electroporation protocol available in this organism. Its use was then extended to fish embryos and cultured mammalian cells.

**Conclusion:**

This versatile vector set opens the way to the mid- to large-scale comparative analyses of protein function and cis-regulatory sequences across chordate evolution. A complete user manual is provided as supplemental material.

## Introduction

The massive increase in sequence and annotation data that can be collected over a short term on any given organism has had major consequences in developmental and evolutionary studies. Besides its impact on classical metazoan model organisms such as mouse, *Drosophila* or *C. elegans*, the genomic revolution also led to the emergence of novel model organisms with interesting phylogenetic positions, and to the molecular revival of classical model organisms that had long been neglected, such as the ascidians. Comparison of the large sets of genomic data in this growing range of species may help understand the emergence of specific traits during evolution. Because of the importance of Gene Regulatory Networks in the generation of organismal complexity [1], the assessment of the level of cis-regulatory sequence activity and protein function during metazoan evolution can be particularly insightful [2], [3].

The chordate phylum groups cephalochordates, tunicates and vertebrates, which share a tadpole-like larval body plan thought to be inherited from their last common ancestor. It is, however, currently unclear to what extent this shared body plan is underlain by common gene regulatory networks. For example, although the tunicates, including the ascidian *Ciona intestinalis*, are thought to be the closest relative to the vertebrates [4], their embryos have a peculiar mode of development based on the existence of a fixed lineage [5]. Further, orthologous ascidian and vertebrate transcription factors can diverge in their activity [6]. Finally, ascidian and vertebrate genomes show poor synteny and little if any conservation of non-coding sequences [7], [8]. Systematic comparison of the activity of ascidian and vertebrate regulatory sequences and protein function may thus help define the extent of conservation in the various chordate developmental GRNs that ultimately lead to the establishment of the same larval body plan.

In this article, we present a set of versatile expression vectors that can be used to systematically compare the activity of proteins and cis-regulatory sequences across the chordate phylum. This vector set is based on the Gateway site-specific recombination cloning technology which allows mid- to high-throughput cloning [9]. In this system entry clones that contain a fragment of DNA of interest flanked by specific attL feet are first generated. Each entry clone can then be recombined with a variety of custom destination vectors that include a selection cassette flanked by attR feet. The resulting expression vectors contain the DNA of interest flanked by attB feet, which are short and expected to interfere minimally with the biological activity of the cloned fragment [10]–[12] . Recent progress provided an expanded collection of recombination sites with distinct specificities, leading to Multisite Gateway technology in which more than one entry clone can be recombined into a destination vector [13]. Multisite Gateway technology has in particular been adapted in the nematode community to make full use of the large sets of promoters (promoterome, [14] ) and predicted Open Reading Frames (ORFeome; [15]) generated in this community. None of these vector systems has however been tested and shown to work in a range of organisms.

**Figure 1:**
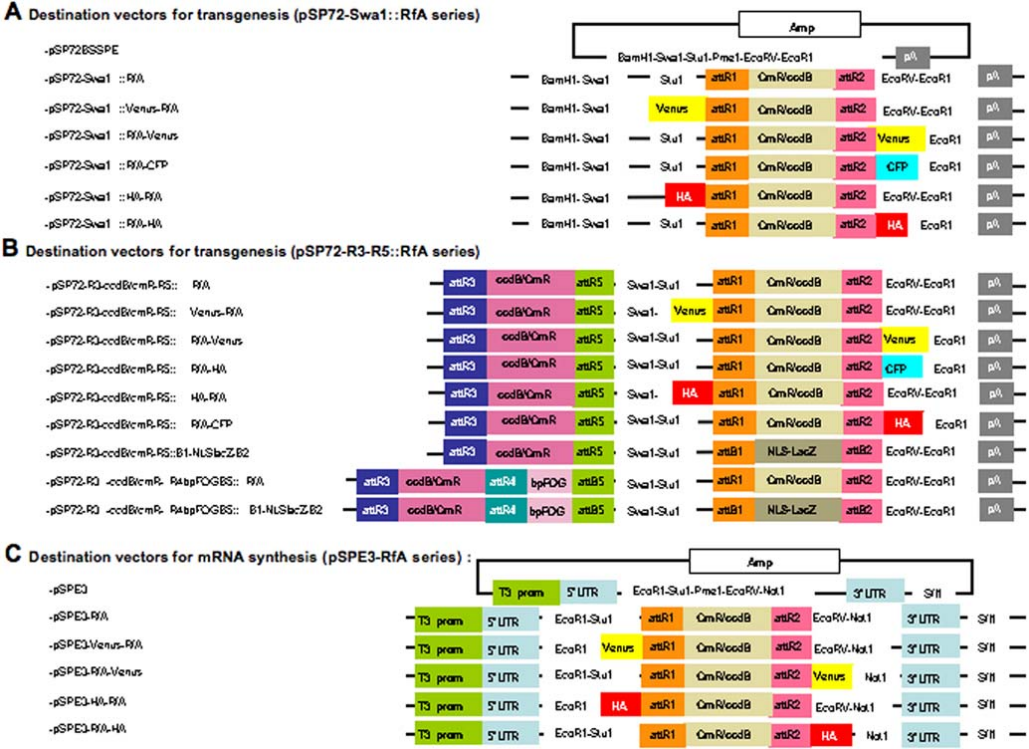
Overview of the vectors described in this article.

The vector set presented here (See Fig. 1 for a general overview) is a general platform which can be used to precisely dissect cis-regulatory sequences, and to overexpress wild type or tagged proteins in a variety of chordate systems. It was developed and first characterised in the embryos of the ascidian *Ciona intestinalis*, in which large scale analyses are easier to perform than in vertebrates, owing to the very efficient embryo electroporation protocol available in this organism [16]. We then showed that the use of these vectors can be extended to both fish embryos and cultured mammalian cell lines. This study thus opens the way for the mid- to large-scale comparative analysis of protein function and cis-regulatory sequences within chordates.

## Results

### Introduction of an RfA cassette into a set of transgenesis vectors

We first adapted the pSP72-1.27 transgenesis vector initially developed for *C. elegans* [17] and also known to be efficient in ascidians [16]. A new polylinker was first inserted between the BamH1 and EcoR1 sites of pSP72-1.27, giving rise to pSP72BSSPE (Fig. 1A, material and methods). This polylinker can be used to clone a Gateway cassette (Pme1), N-terminal (Stu1) or C-terminal (EcoRV) tags and a driver (Swa1).

As in most Gateway cDNA libraries, including available *Ciona* Gateway cDNA libraries, the cDNA inserts are placed between attL1 and L2 recombination sites, we used an attR1/R2 destination cassette compatible with these inserts. Three types of attR1/R2 cassettes, RfA, RfB and RfC, exist differing in the reading frame to be used when designing the entry clones to respect the frame of the N- and C-terminal tags. To enforce compatibility between entry clones generated by the various laboratories of a scientific community, all our vectors make use of the RfA cassette, which is compatible with the Invitrogen Gateway bacterial expression vectors and Proquest Yeast 2 hybrid system (pDest22 and 32 vectors). Thus, the ORF entry clones used for *in vivo* tests with this vector set can be used for additional characterisation of the function of proteins of interest.

Functional analysis of a cDNA of interest includes the determination of its subcellular localisation by overexpression of a fluorescently tagged protein. For this, we introduced an N-terminal tag preceded by a Kozak sequence, or a C-terminal tag followed by a Stop codon in the Stu1 and EcoRV sites, respectively, placed on either side of the cassette. The current vector set includes N- and C-terminal Venus YFP and HA tags as well as C-terminal CFP (Fig. 1A).

**Figure 2:**
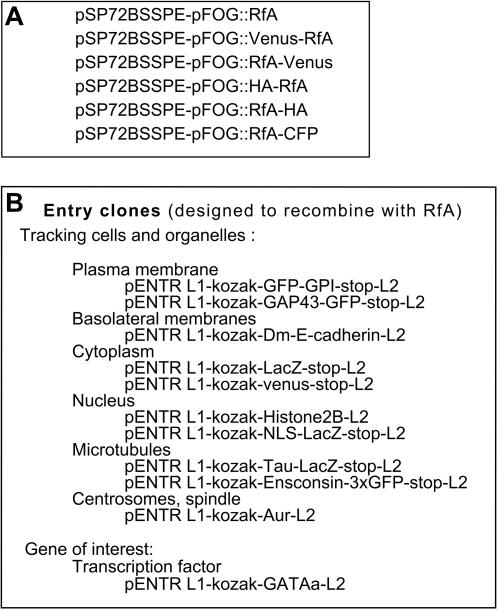
Destination vectors and ORF entry clones used to test the pSP72-swa1::RfA vector series. A) Destination vectors for the overexpresion of WT or tagged ORFs under the control of the animal-specific cis-regulatory regions of *Ciona Friend of GATA (Ci-FOG).* B) Generated ORF entry clones with the obeserved subcellular localisation of the corresponding tagged proteins.

To test the functionality of these vectors, the Ci-FOG (*Ciona* Friend Of Gata) cis-regulatory sequences, driving expression in all *Ciona* animal blastomeres between the 16-cell and early gastrula stages [18], was cloned into the Swa1 site of several vectors of the pSP72BSSPE swa1::RfA series, giving rise to the pSP72BSSPE-pFOG::RfA series of destination vectors (Fig. 2A). We next generated a collection of entry clones for a set of ORFs of interest including markers expected to mark specific cell compartments including the nucleus, plasma membrane, basolateral membranes, whole microtubule network and centrosomes (Figure 2B). A detailed procedure for generating ORF entry clones can be found on page 11 of the accompanying Chordate Gateway vector manual (Manual S1).

**Figure 3:**
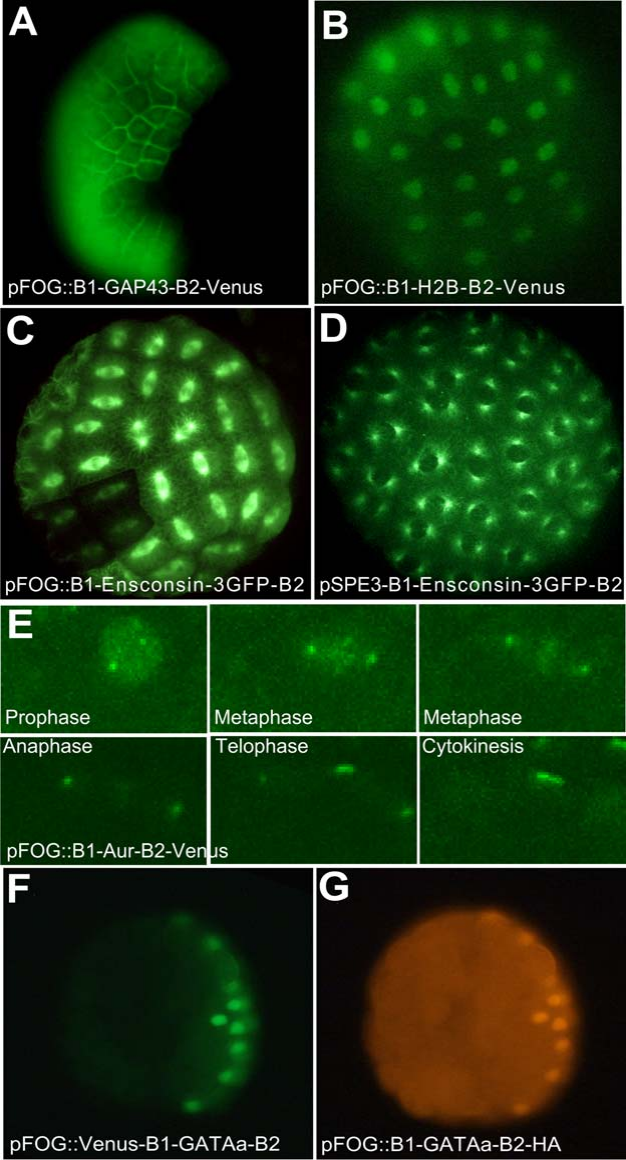
Subcellular localisation of tagged proteins expressed from pSP72-pFOG::RfA vector series. A, B, F, G epifluorescence pictures of embryos expressing GAP-43 venus (A), Histone2B-venus (B), venus-GATAa (F) and GATAa-HA (G). F and G show the same embryo co-electroporated with pSP72-pFOG::Venus-B1-GATAa-B2 and pSP72-pFOG::B1-GATAa-B2-HA. C, D, E projections of confocal stacks of images from embryos either electroporated (C, E) or microinjected with mRNA (D) and expressing ensconsin-3GFP (C, D), or ci-aurora kinase (E). Due to the mosaic expression of the transgene, some cells express the marker at a lower level (C, F, G).

These entry clones were recombined with pSP72BSSPE-pFOG::RfA destination vectors (Fig 2A) and the resulting expression clone were electroporated into fertilised ascidian eggs. Overexpression of these proteins had no effect on embryo viability. Fig. 2B recapitulates the observed subcellular localisation of the produced fluorescent protein. Fig. 3 illustrates some of the results obtained. At the early gastrula stage, pSP72-pFOG::B1-GAP43-GFP-B2 targeted GFP expression to the cell membrane of animal cells (Fig. 3A) [19], [20]. pSP72-pFOG::B1-Kozak-Histone2B-B2–Venus decorated the animal nuclei (Fig. 3B). pSP72-pFOG::B1-Ensconsin-3GFP-B2 marked the whole microtubule network (Fig. 3C). Interestingly, localisation of the venus-tagged single *Ciona* Aurora kinase, Ci-Aur (Fig. 3E), driven by pSP72-pFOG::B1-aurora-B2-Venus recapitulated the cellular localisation of the two well-studied vertebrate aurora kinases Aurora A and Aurora B during mitosis. In prophase, Ci-Aur was found, like vertebrate Aurora A, around the centrosomes and, like Aurora B, in a punctate staining in the nucleus, which likely corresponds to a chromosomal centromeric staining. During metaphase, Ci-Aur-Venus strongly localised to the centrosome and uniformly on the mitotic spindle. In anaphase and telophase, the spindle staining concentrated around the spindle midzone, like vertebrate Aurora B [21] .During cytokinesis, spindle midzone/midbody staining increased, whereas centrosomal staining disappeared.

Taken together, these results indicate that the introduction of the polylinker and Gateway cassette into the pSP72 vectors does not significantly interferes with the subcellular localisation of the expressed proteins. Likewise, the expected animal restriction of the domain of expression of the fluorescent proteins, suggests that the introduction of the attR1/R2 Gateway recombination sequences does not alter the activity of the cis-regulatory regions used as drivers. The suitability of the pSP72BSSPE-Swa1::RfA series of transgenesis vectors to drive expression of functional transcription factors or signalling ligands of interest under control of various cis-regulatory regions was further recently reported [18], [22].

Finally, co-electroporation of pSP72-pFOG::B1-Kozak-GATAa-B2-HA and pSP72-pFOG::Venus-B1-GATAa-Stop-B2, and immunodetection with antibodies against HA or GFP respectively, illustrates that co-electroporation of two similar vectors is an efficient way to co-express several proteins in the same cells (Fig. 3F, G).

### Design of 2-cassette transgenesis vectors in which both cis-regulatory sequences and ORFs can be Gateway cloned

Although the transgenesis vectors presented in the first section offer significant improvement over the parental pSP72 series, the cis-regulatory regions still need to be conventionally cloned precluding the scaling up of regulatory sequence analyses. We next transformed these vectors to allow Gateway cloning of both cis-regulatory sequences and ORF of interest. Previous work indicated that flanking a cis-regulatory region with attB1 and attB2 recombination feet does not interfere with its activity in E. coli, insect (Sf9 line), or mammalian cells [9], a feature we confirmed in ascidians [22]. However, because attB1 and attB2 recombination sites were already used to clone the ORFs in our vectors, and because no suitable cassette was available, we developed a novel Gateway R3-ccdB-CmR-R5 cassette (see below) to receive cis-regulatory regions, and inserted it into the Xho1-Xba1 sites upstream the BamH1 site of vectors of the pSP72BSSPE swa1:: RfA series described in the previous section (Fig. 1B). This cassette recombines with pEntr-L3-cis-reg-L5 entry clones containing cis-regulatory regions.

**Figure 4:**
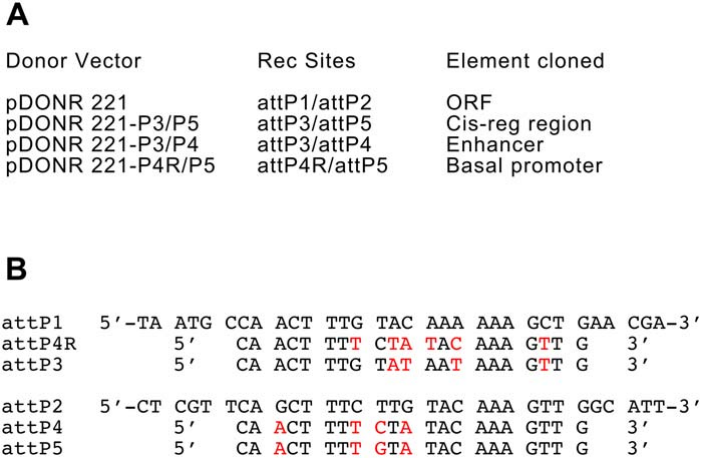
Donor vectors of different recombination specificities. A) list of generated vectors with the identity of the recombination sites and type of element cloned. B) Sequence of the recombination sites used in the different donor vectors.

To create the novel Donor/Destination system based on the attB3-B5 recombination sites we first constructed the pDONR-221-P3/P5 donor vector (Fig. 4A). For this, point mutations were introduced into the 7 nucleotides of the attP1 and attP2 recombination sites of pDONR-221 that confer the specificity of the recombination (Fig. 4B) [23]. The resulting vector was tested by amplifying the pFOG regulatory region and flanking them with attB3 and attB5 recombination sequences. Recombination in a BP reaction of this 2 kb fragment in pDONR-221-P3/P5, followed by transformation into electrocompetent DH5alpha bacteria produced several hundred colonies (Table 1). Sequence analysis of 13 colonies revealed that the recombination was correct in all tested colonies. Additional cloning of the muscle-specific Ci-Sna cis-regulatory region (pSna; [24] ) confirmed the high efficiency of BP cloning into this new donor vector (not shown).

**Table 1:** REACTION EFFICIENCY AND SPECIFICITY

BP Reaction (attB PCR product X pDonor)	Nber Bacterial colonies*	Nber of Colonies analysed	Accurately recombined clones	Percentage of correct pEntry
B1-kozak-Venus-stop-B2 **X** pDONR 221	>1000	3	3	**100%**
B3-pFOG-B5 **X** pDONR P3-P5	>300	13	13	**100%**
B3-a-element-B4 **X** pDONR P3-P4	>1000	3	3	**100%**
B4R-bpFOG-B5 **X** pDONR P4R-P5	>500	20	2	**10%**
**LR reaction (pEntry X pDestination)**	**Nber Bacterial colonies ** *	**Nber of Colonies analysed**	**Accurately recombined clones**	**Percentage of correct Expression clones**
pENTR attL1-kozak-Venus-stop-L2 **X** pSP72-pFOG-RfA	>1000	5	5	**100%**
pENTR attL3-pFOG-attL5 **X** pSP72-R3-R5::B1-kozak-NLS-LacZ-stop-B2	>500	3	3	**100%**
pENTR attL3-pFOG-attL5 **X** pSP72-R3-R5::R1-kozak-GATAa-R2-Venus	>150	5	5	**100%**
pENTR attL3-pFOG-attL5+pENTR attL1-kozak-GATAa-attL2 **X** pSP72-R3-R5::RfA-Venus	<20	5	4	**80%**
pENTR-L3-a-element-L4+pENTR-R4-bpFOG-L5 **X** pSP72-R3-R5:: B1-kozak-GATAa-B2-Venus	<20	4	4	**100%**
pENTR-L3-a-element-L4+pENTR-R4-bpFOG-L5 **X** pSP72-R3-R5::B1-kozak-NLS-LacZ-stop-B2	<20	4	3	**75%**

* Nber of colonies obtained by plating the whole recombination reaction following electrotransformation into DH5α bacteria (>10^8^ col/µg)

**X**: means recombination between attB PCR product and pDonor or pEntry and pDestination.

The destination R3-ccdB-CmR-R5 destination cassette was generated from this donor vector by orienting the Gateway BP reaction, which generates both an attL-flanked entry construct and an attR-flanked destination cassette, so that the destination cassette is the cloned product of the recombination [23].

**Figure 5:**
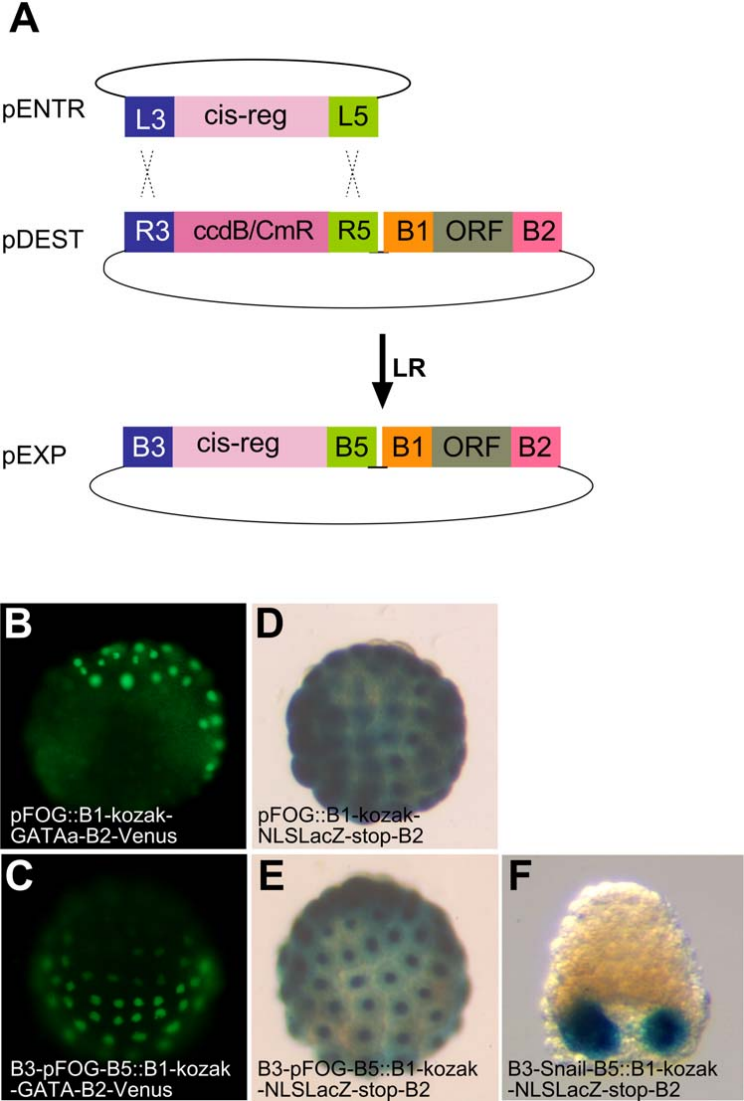
Cloning of cis-regulatory sequences in the R3-R5 cassette. A) scheme of the recombination of an L3-cis-reg-L5 entry clone into the R3-R5 cassette of a destination vector. B-F) Activity of the indicated constructs introduced in embryos by electroporation. B, C) The reporter was GATAa-venus. D-F) The reporter was NLS-LacZ.

To test the functionality of this cassette, it was cloned between the Xho1 and Xba1 sites of pSP72 swa1::B1-GATAa-B2-Venus and pSP72 swa1::B1-Kozak-NLS-LacZ-stop-B2, thus giving rise to the pSP72-R3-ccdB-CmR-R5::B1-Kozak-GATAa-B2-Venus pSP72-R3-R5::B1-Kozak-NLS-LacZ-stop-B2 destination vectors. The efficiency of LR recombination of cis-regulatory sequences into these new vectors, as schematised on Fig. 5A, was assayed by recombination with the pENTR-attL3-pFOG-attL5 entry clone obtained above. The number of ampicillin-resistant colonies obtained was comparable to what is typically obtained with the R1-R2 system (Table 1). Following transformation into bacteria, 3 to 5 colonies were analysed by restriction digest and sequencing, revealing correct recombination in all cases (Table 1). To test the specificity of the attR3-R5 recombination, an LR reaction was performed between the same amount of pENTR-attL3-pFOG-attL5 and an RfA (attR1/R2) destination vector. Following transformation into bacteria, no ampicillin-resistant colony were obtained, indicating that attR3-R5 recombination is hihgly specific as attL3/L5 entry clones recombine into attR1/R2 destination cassettes at least two orders of magnitude less efficiently than into attR3/R5 cassettes (not shown). Similarly, recombination of an attL1-L2 entry clone into a vector bearing an R3-R5 cassette was very inefficient (not shown).

We next tested whether introduction of the exogenous attB3/B5 feet interfered with the activity of the cloned driver. We electroporated in parallel pSP72-B3-pFOG-B5::B1-Kozak-GATAa-B2-Venus and pSP72-pFOG::B1-Kozak-GATAa-B2-Venus in which the cis-regulatory sequences of pFOG was inserted by standard restriction enzyme-mediated cloning. The results obtained with these two vectors were indistinguishable both in terms of the identity of the cells expressing the fluorescently tagged GATAa protein, and of the level of expression of the protein (Fig. 5B, C). Identical results were also obtained when comparing Xgal staining in parallel electroporations of pSP72-pFOG::NLS-lacZ with pSP72-B3-pFOG-B5::B1-Kozak-NLS-lacZ-stop-B2 (Fig. 5D, E). Likewise, electroporation of pSP72-B3-pSna-B5::B1-Kozak-NLS-lacZ-stop-B2 led to the expected muscle specific X-gal staining (Fig. 5F), further indicating that combinations of attB1, B2, B3 and B5 do not interfere with the activity of transgenic constructs in ascidian embryos.

The attR3/R5 recombination cassette thus provides an alternative to the classical attR1/R2 Gateway system in terms of specificity of recombination, and lack of interference with the transcriptional activity of cloned drivers.

### Simultaneous introduction of a cis-regulatory region and an ORF of interest in a 2-cassette destination vector

**Figure 6:**
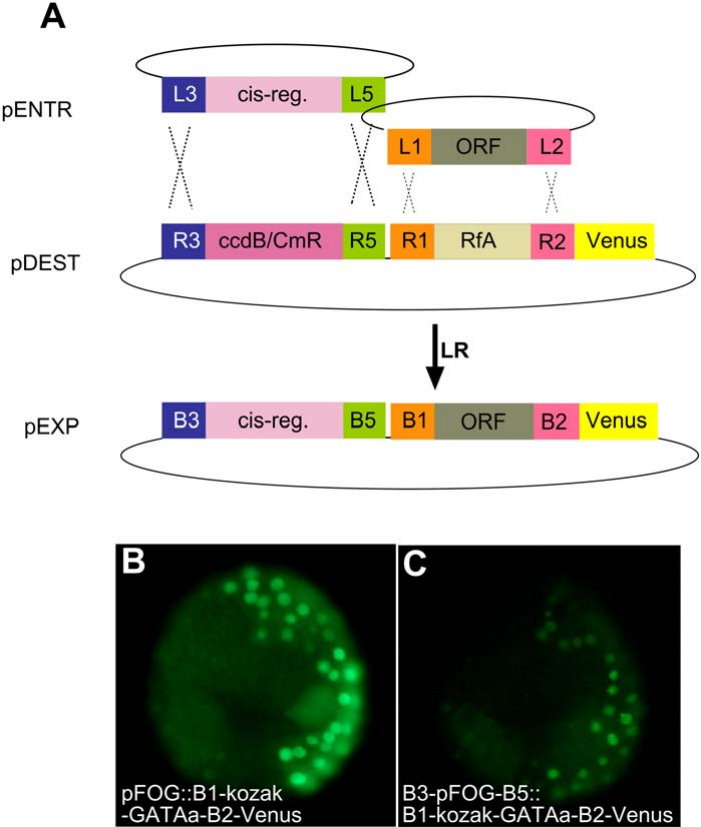
Simultaneous introduction of a cis-regulatory region in the R3-R5 cassette and an ORF in the RfA cassette. A) Experimental scheme. B) Comparison of the activity by electroporation of the indicated constructs. The weaker fluorescence observed in C is not representative. Due to mosaic expression of the transgene, only some cells express the fluorescent marker.

We next wanted to test whether simultaneous recombination of a cis-regulatory region and ORF could be achieved (Fig. 6).

We cloned the attR3-ccdB-CmR-attR5 cassette into the Xho1-Xba1 sites of pSP72-Swa::RfA-Venus, giving rise to the pSP72-R3-ccdB-CmR-R5::RfA-Venus two-cassette destination vector (Fig. 1B). We then simultaneously LR recombined into this destination vector the pENTR attL3-pFOG-attL5 cis-regulatory entry clone and the pENTR attL1-Kozak-GATAa-attL2 ORF entry clone (Fig. 2). Electro-transformation into DH5a bacteria gave rise to one order of magnitude fewer colonies than the recombination of a single attL3-L5 or attL1-L2 entry clone (Table 1). Analysis by restriction enzyme digestion of 5 colonies, revealed that 4 of these colonies contained a plasmid of expected structure, demonstrating a recombination specificity of 80% similar to the specificity obtained with a single recombination involving either R1/R2 or R3/R5. Electroporation of the resulting plasmids confirmed the functionality of the recombined vectors (Fig. 6B, C).

Encouraged by these results, we introduced the R3-R5 cassette into the Xho1-Xba1 sites of several electroporation destination vectors for analysis of untagged, C-terminally or N-terminally tagged proteins under control of cis-regulatory regions of interest (Fig. 1B).

### Replacement of a single segment in a pSP72-B3-cis-reg-B5::B1-ORF-B2 expression clone

While the presence of the two cassettes greatly enhances the flexibility of the vector system, simultaneous recombination of two cassettes into a destination vector, which is less efficient than simple recombination, is often not necessary. For instance, scientists interested in analysing the activity of cis-regulatory sequences may simply want to recombine various genomic fragments into a destination vector that places them in front of a suitable reporter gene. Conversely, a laboratory interested in the formation of a given tissue, may want to place a variety of ORFs under control of a driver specific for this tissue. In this section, we show that starting from a pSP72-B3-cis-reg-B5::B1-ORF-B2 expression clone, it is possible to free either the attR3/R5 or the RfA cassette from the expression clone, thus giving rise to pSP72-R3-ccdB-CmR-R5::B1-ORF-B2 or pSP72-B3-cis-reg-B5::RfA single-cassette destination vectors.

**Figure 7:**
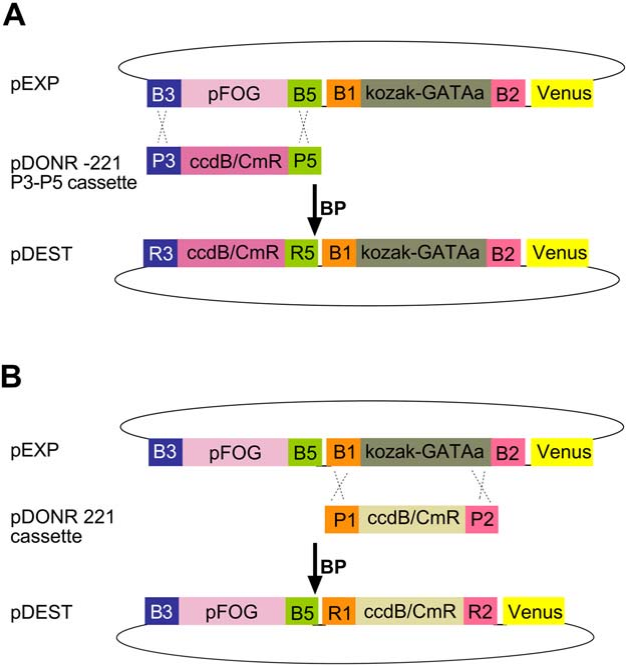
Strategy for the replacement of a cis-regulatory (A) or ORF ((B) segment by the corresponding Gateway cassette.

To exemplify the process, we started from the pSP72-B3-pFOG-B5:: B1-Kozak-GATAa-B2-venus expression clone. Two BP reactions were set between the expression clone and either an attP3/P5 (Fig. 7A) or an attP1/P2 cassette (Fig. 7B) purified from the corresponding donor vector (see materials and methods). Following transformation into ccdB-resistant DB3.1 cells, transformants were selected on Ampicillin+Chloramphenicol plates to isolate pSP72-B3-pFOG-B5::RfA-Venus and pSP72-R3-ccdB-CmR-R5::B1-Kozak-GATAa-B2-Venus. The efficiencies of these reactions were comparable to that of the introduction of an entry clone into the attR1-ccdB-CmR-attR2 destination cassette. In the case of pSP72-B3-pFOG-B5::RfA-Venus, 4 out of 5 colonies analysed by restriction enzyme digest had the expected structure and could recombine with a different attL1/L2 entry clone (not shown). A similar efficiency and specificity was obtained for pSP72-R3-ccdB-CmR-R5::B1-GATAa-B2-Venus.

This strategy can be generally applied and these intermediate destination vectors can constitute tools of general interest. For instance, the pSP72-R3-ccdB-CmR-R5::B1-Kozak-NLS-LacZ-Stop-B1 vector of the current release allows to clone cis-regulatory sequences in front of NLS-lacZ reporter.

### Independent analysis of basal promoters and distal cis-regulatory modules using a Multisite Gateway strategy

**Figure 8:**
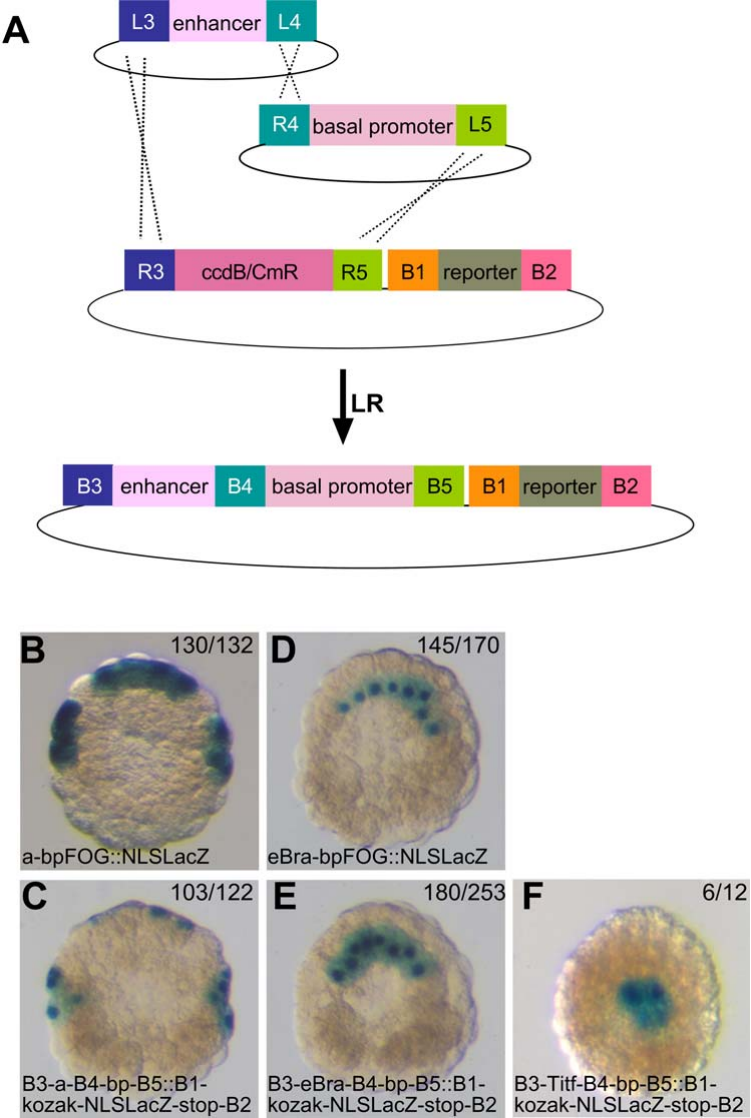
Simultaneous introduction of an enhancer and basal promoter into a destination vector. A) Experimental scheme. B-F) Results of the electroporation of the indicated constructs generated by conventional cloning (B, D) or by Gateway cloning (C, E, F). The numbers indicate the proportion of embryos with the shown lacZ staining.

Cis-regulatory sequences are commonly composed of basal promoters located close to the start of transcription, and tissue-specific enhancers or silencers located at a distance. Analysis of a cis-regulatory logic requires that these two types of modules be analysed, and hence cloned, separately. We thus adapted the Gateway Multisite technology [13] to independently recombine two entry clones, one carrying a basal promoter, the other a distal element, into the attR3/R5 destination cassette of our vector set (Fig. 8A). For this, we manipulated the attP1 and P2 recombination sites to create two new donor vectors, pDONR-221-P3/P4, used to clone the distal element, and pDONR-221-P4R/P5, used to clone the basal promoter (see materials and methods) (Fig. 4A, B).

To test the pDONR-221-P3/P4 vector, we amplified by PCR and flanked with attB3 and B4 sites the early neural element of the *Ciona intestinalis Ci-Otx* gene (a-element, [25] (see materials and methods and Manual S1 page 16 for the design and sequence of the PCR primers). BP recombination of this fragment with pDONR-221-P3/P4, followed by transformation gave rise to a similar number of colonies as a classical B1-B2 recombination into pDONR-221 (Table 1). 3 colonies were picked at random and analysed by restriction digest and sequencing. All 3 had the structure expected for pENTR-L3-a-element-L4. Entry clones for the early notochord eBra enhancer ( [16]; pENTR-L3-eBra-L4) and the endodermal enhancer of Ci-Titf1 ( [26]; pENTR-L3-Ci-Titf-L4) were similarly generated.

To test the pDONR-221-P4R/P5 vector, we PCR amplified the basal promoter of the FOG gene (bpFOG; [22] ), and flanked it with attB4R and attB5 sequences. This fragment was BP recombined into pDONR221-P4R/P5. The efficiency of this reaction, as measured by the number of kanamycin-resistant colonies obtained, was high (Table 1). Restriction enzyme digest analysis revealed, however, the presence of two populations of plasmids. Plasmids from 2 out of 20 colonies analysed showed the restriction digest profile expected for pENTR-attR4-bpFOG-L5. The remaining plasmids appeared to be empty. This reduced specificity is not a serious problem. As the number of colonies obtained is not limiting, PCR selection of the correctly recombined versus empty clones is simple, and the number of basal promoters routinely used to characterise the activity of distal elements limited. Yet, we wanted to further understand why a large proportion of recombined clones had undergone an illegitimate recombination. We thought that the sequence homologies between attP4R and attP5 may allow recombination between these attP sites. To test this hypothesis, we set up a BP recombination reaction with pDonor-attP4R/P5 alone, in absence of any attB4R/B5 PCR product. The result of this reaction was transformed into DH5α and plated onto Kanamycin plates. A high number of clones was obtained and sequence analysis confirmed the illegitimate recombination between attP4R and attP5, resulting in a loss of the ccdB and chloramphenicol resistance genes.

We next simultaneously recombined pENTR-L3-a-element-L4 and pENTR-R4-bpFOG-L5 into the attR3/R5 Gateway cassette of either pSP72-R3-ccdB-CmR-R5::B1-GATAa-stop-B2-Venus or pSP72-R3-ccdB-CmR-R5::B1-Kozak-NLS-LacZ-stop-B2, according to the scheme presented Fig. 8A. 10 femtomoles of each plasmid were incubated with the LR Plus clonase mix and the result transformed into electrocompetent DH5α bacteria (2.2 10^8^ colonies/µg of plasmid DNA). The results presented in Table 1 demonstrated that while only few colonies were obtained, over 80% of these colonies harboured a plasmid of expected structure.

In many cases, simultaneous recombination of a minimal promoter and an enhancer is however not necessary, most enhancers being tested in the context of a very small number of minimal promoters.

By inverting as previously the BP reaction between the P3-ccdB/CmR-P4-DONR cassette and the pSP72-B3-a-element-B4-bpFOG-B5-B1-Kozak-NLS-LacZ-stop-B2 expression clone, we generated the pSP72-R3-ccdB-CmR-R4-bpFOG-B5::B1-Kozak-NLS-LacZ-stop-B2 destination vector. This construct is useful to test enhancer sequences and is included in the current vector collection release. Functionality of this vector was further demonstrated by recombining it with the pENTR-L3-eBra-L4 and pENTR-L3-Ci-Titf-L4 enhancer clones constructs.

Finally, we tested whether the insertion of attB3, B4 and B5 interfered with the activity of the cloned regulatory sequences. The three expression clones pSP72-B3-a-element-B4-bpFOG-B5::B1-NLS-LacZ-B2, pSP72-B3-eBra-B4-bpFOG-B5::B1-NLS-LacZ-B2, and pSP72-B3-Ci-Titf-B4-bpFOG-B5::B1-NLS-LacZ-B2 were electroporated into fertilised eggs and their activity measured by Xgal staining at the 110-cell (a-element, eBra) and mid-gastrula (Ci-Titf) stages. The presence of X-gal positive cells was in all cases restricted to the expected tissues, induced neural precursors for the a element, notochord for eBra and endoderm for Ci-Titf, establishing that the attBs do not alter the spatial activity of the enhancers (Fig. 8 C, E, F).

To test whether attBs altered the level of activity of the enhancers, we electroporated in parallel a construct generated via Gateway technology (pSP72-B3-a-element-B4-bpFOG-B5::B1-NLS-LacZ-B2, pSP72-B3-eBra-B4-bpFOG-B5::B1-NLS-LacZ-B2) and the corresponding construct generated by conventional restriction enzyme cloning (pSP72-a-element-bpFOG::NLS-LacZ, pSP72-eBra-bpFOG::NLS-LacZ). Analysis of LacZ activity at the 110-cell stage by X-gal staining revealed a similar level of activity, the presence of the attB sequences leading to a 15-20% lower level of expression unlikely to interfere with most applications (Fig 8 B-E).

Taken together these experiments demonstrate that the B3-B4-B5 Multisite Gateway strategy allows to simultaneously or sequentially clone an enhancer and a basal promoter into pSP72 R3-R5::B1-ORF-B2 destination vectors and that the presence of attB sequences does not significantly affect the activity of these elements.

### Generation of a set of vectors to produce synthetic mRNAs for microinjections

Microinjection of synthetic mRNAs is an alternative route to overexpress proteins in chordate embryos. To generate a set of synthetic mRNA vectors compatible with the same entry clones as the electroporation vectors, we adapted the pRN3 synthetic mRNA vector, initially developed for Xenopus studies [27] and used in ascidian [25], urchin [28] and *zebrafish* embryos [29]. A novel polylinker was introduced between the EcoR1 and Not1 sites of pRN3 and used to clone the RfA Gateway cassette (Pme1) and flanking N- (Stu 1) or C-terminal (Eco RV) fusion tags (Fig. 1C). These mRNA vectors were designed to be compatible with the ORF entry clones generated for the electroporation vectors.

To test the functionality of the vectors, we recombined pEntr-L1-Kozak-Ensconsin-3GFP-Stop-L2 into pSPE3-RfA to give rise to expression clones, which were used to synthesize mRNA *in vitro*. Fig. 3D illustrates that the resulting mRNA, when injected into ascidian eggs efficiently drives production of a protein, which localises as expected to the microtubule network.

### Use in vertebrate embryos and cells

Finally, we tested whether the Gateway transgenesis vectors would also be suitable for use in vertebrate systems.

**Figure 9:**
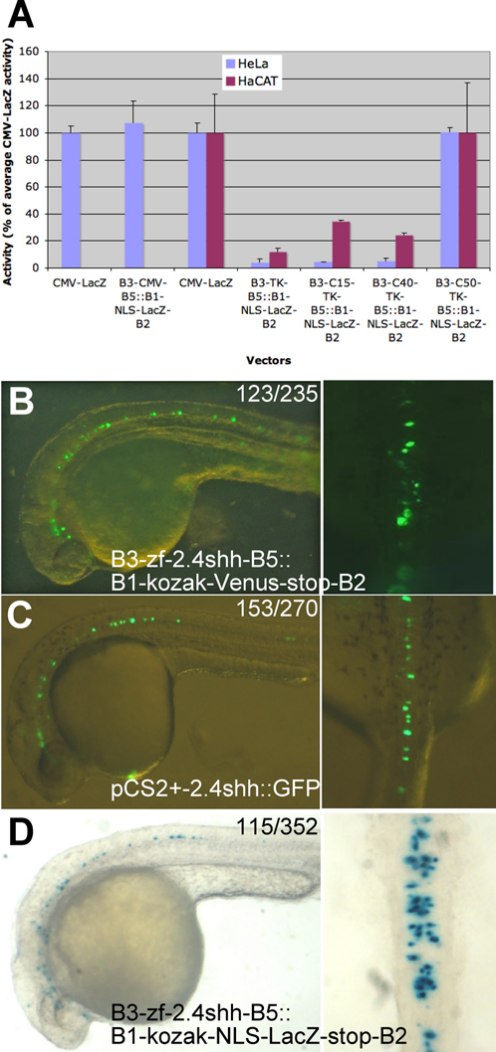
Chordate expression constructs are suitable for mammalian cells and *zebrafish* embryos. A) Transfection in mammalian cells of the indicated murine enhancers. The Y-axis shows the percentage of activity (% of LacZ positive cells) of tested Gateway-cloned enhancers relative to a control CMV-lacZ construct. The left most two columns were obtained in independent experiments from the other columns, restricted to Hela cells. B-D) Transient transgenesis by microinjection into *zebrafish* eggs of the indicated constructs. Left panel, lateral view, right panel, dorsal view, of 30 hour post-fertilization stage *zebrafish* embryos. No change in the intensity or localisation is observed when Gateway expression constructs are used (B, D) instead of pCS2+-based constructs (C). The numbers indicate the proportion of embryos with the shown lacZ or GFP staining.

To test whether the pSP72-R3-R5::RfA vectors were suitable to monitor enhancer activity in mammalian tissue culture, we first constructed a Gateway entry clone containing the strong ubiquitous CMV enhancer in front of the tk promoter (pENTR-attL3-CMV-tk-attL5), LR recombined it into pSP72-R3-ccdB-CmR-R5::B1-Kozak-NLS-LacZ-Stop-B2. The activity of this construct in HeLa carcinoma cells was very similar to that of pCMV-LacZ, obtained by classical restriction enzyme-mediated cloning (Fig. 9A, left-most two columns). Next, we tested whether the Gateway system interfered with tissue specific expression. Three entry clones were generated containing the minimal tk promoter (pENTR-attL3-tk-attL5) [30], the murine p63 C15 enhancer [31] in front of the tk promoter (pENTR-attL3-C15-tk-attL5), the murine p63 C40 enhancer [31] in front of the tk minimal promoter (pENTR-attL3-C40-tk-attL5) and the murine p63 C50 enhancer placed in front of the tk promoter (pENTR-attL3-C50-tk-attL5). C15 and C40 were previously reported to be keratinocyte-specific [31], while C50 is a strong ubiquitous enhancer (Dario Antonini and CM, unpublished). Expression constructs, generated by LR recombination of the resulting entry clones into pSP72-R3-ccdB-CmR-R5::B1-Kozak-NLS-LacZ-Stop-B2, were transfected into HeLa cells or into HaCaT keratinocytes and the number of LacZ positive cells analysed (Fig. 9A). pSP72-B3-tk-B5::B1-Kozak-NLS-LacZ-Stop-B2 had a very low level of activity in both cell lines in keeping with the idea that no exogenous enhancer was present in the vector backbone. pSP72-B3-C50-tk-B5::B1-Kozak-NLS-LacZ-Stop-B2 drove a strong expression of the reporter in both cell lines, as expected. The activity of pSP72-B3-C15-tk-B5::B1-Kozak-NLS-LacZ-Stop-B2 and pSP72-B3-C40-tk-B5::B1-Kozak-NLS-LacZ-Stop-B2 was weaker than that of the C50 construct and restricted to the keratinocytes as expected.

To further assess that the vector backbone and Gateway recombination sequences did not interfere with the activity of vertebrate cis-regulatory regions, the 2.4kb *zebrafish* shh upstream cis-regulatory region, driving expression in the anterior floor plate of fish embryos [32], [33] were amplified with flanking attB3-B5 feet and recombined into pDONR-221-P3/P5. The resulting entry clone was jointly recombined with pENTR-L1-Kozak-Venus-Stop-L2 into pSP72-R3-ccdB-CmR-R5::RfA to generate pSP72-B3-zf-2.4shh-B5::B1-Kozak-Venus-Stop-B2. This construct was microinjected into *zebrafish* fertilised eggs (Fig. 9B) alongside a similar construct (pCS2+-shh2.4::gfp) (Fig. 9C) in a commonly used fish transgenesis vector lacking the Gateway recombination sequences, pCS2+ [32]. The distribution and number of transiently transfected fluorescent cells and the number of expressing embryos at 1 day post-fertilisation was indistinguishable with the two vectors (pCS2+-2.4shh:GFP: 56,7% of injected embryos were expressing the GFP; pSP72-B3-zf-2.4shh-B5::B1-Kozak-Venus-Stop-B2: 52,3% showed YFP-expression (Fig. 9B, C). Likewise, distribution of LacZ positive cells in embryos injected with pSP72-B3-zf-2.4shh-B5::B1-Kozak-NLS-LacZ-Stop-B2 was similar to that obtained with the fluorescent reporter (32,7% of injected embryos showed lacZ-positive cells) (Fig. 9 D). The vector set described in this article can thus be used throughout the chordate phylum.

## Discussion

In this article, we presented a collection of Gateway vectors, initially developed for *Ciona* embryos, but also functional in *zebrafish* embryos, and mammalian cells in culture. The collection includes two types of vectors, compatible with the same collection of ORF entry clones. The pSPE3 series allows to synthesize *in vitro* mRNA for wild type or tagged proteins of interest. The pSP72 series of transgenesis/electroporation vectors allows to simultaneously recombine a cis-regulatory region and an ORF of interest into a two-cassette vector. Further, we showed that a cloned fragment of cis-regulatory or ORF DNA can be remobilised to generate a novel destination vector, which can in turn be used to recombine one or two new DNA segments. Although several other Gateway eukaryotic expression vector sets have been reported in the literature, designed for *C. elegans* [10], *Drosophila* (http://www.ciwemb.edu/labs/murphy/Gateway%20vectors.html), plants [34], [35] or mammalian cells [12] our vector set represents to our knowledge the only vector set that allows to test the activity of complex cis-regulatory sequences, enhancers, promoters as well as the function and subcellular localisation of the product of ORFs. These vectors, as well as a collection of entry clones for *Ciona* cis-regulatory regions and fluorescent reporters for major sub-cellular organelles, are released to the community as a 96 well-plate stock, accompanied with a complete user manual.

In this discussion, we highlight the flexibility of the system and present its foreseeable applications to the systems biology of chordate embryonic development.

### Gateway recombination feet do not interfere with protein or cis-regulatory activity

The aim of this work was to generate a versatile vector suite allowing the mid- to high-throughput analysis of cis-regulatory sequences and protein function. We chose to adopt the Gateway system to bypass the limitations of restriction enzyme mediated cloning. In particular, restriction enzyme mediated cloning of an insert requires that the cloning enzymes do not cut within the insert. This cloning strategy is insert-dependent, precluding systematic cloning approaches. In contrast, the Gateway system provides an insert-independent cloning strategy [36]. A possible downside of this system, however, is that, while introducing short restriction enzyme sites is unlikely to interfere with the activity of cis-regulatory elements or the translation of ORFs, the 25 bp attB sequences may introduce a bias. As previously found in E. coli, insect and mammalian cells, we did not detect a decrease in the translation of proteins translated from ORFs including attB1 or B2 sequences. An interference with the sub-cellular localisation of tagged proteins is more difficult to detect as addition of N- or C-terminal tags is known to sometimes interfere with the localisation of the fusion protein [37]. We note, however, that using our vectors in *Ciona* embryos, we could obtain fusion proteins including translated attB sequences and localising to the basolateral membranes (Dm-E-Cadherin) [38] , nucleus (Histone H2), cytoplasm (Venus), and centrosomes (Aurora kinase) as expected for the fusion protein that was tracked. This suggests that the few amino acid encoded by attB1/B2 recombination sites do not significantly interfere with protein localisation.

Likewise, although attB3, B4 or B5 recombination feet could have included specific binding sites for transcription factors that could modify the activity of cloned cis-regulatory regions, we detected no significant effect in *Ciona* or *zebrafish* embryos or tissue culture cells.

### Relationships and compatibility with other Gateway vector systems: from in vivo activity to biochemical characterisation

When designing the system, we took care to make it compatible with existing systems. The choice of an attR1-attR2 Gateway cassette to receive the ORFs was motivated by the fact that most sequenced Gateway cDNA libraries, as well as *C.elegans* and Human ORFeomes were constructed in an attP1-attP2 donor vector. The choice of the RfA among the available attR1-attR2 cassettes makes our ORF entry clones compatible with these ORFeomes. In addition, the RfA cassette is also used in the Proquest Y2H system to look for protein interactors, and in the Invitrogen bacterial expression vectors. Thus, ORFs characterised by overexpression in vivo, can be readily transferred into bacterial or yeast expression vectors that allow further biochemical characterisation and interaction studies.

Few studies have made use of the multisite Gateway system to analyse cis-regulatory regions, possibly because the Gateway system is ideally suited for mid- to large-scale studies and that few model organisms allow such analyses. In *C. elegans*, a different cassette bearing attR4 and attL1 sites was used to build a genome-wide collection of 5′ Gene flanking regions, the promoterome [14]. This cassette is not compatible with our attR3/R4/R5 cassettes, but, unlike the activity of ORFs [39], the activity of cis-regulatory sequences is not likely to be conserved between nematodes and chordates, as conservation between worms and fly, two protostomes, is already very limited [40].

### Opening the way to a systems biology approach to *Ciona* development

The recent sequencing of several vertebrate and invertebrate chordate genomes has widened the gap between the identification and the functional characterisation of genes. In a majority of cases, the effects of a gene gain- or loss-of-function in specific tissues is unknown, as is the subcellular localisation of the gene product, or identity of the regulatory elements that drive its expression. Development of the current vector set aimed at facilitating the large scale unravelling of protein and cis-regulatory sequence activity.

The vector set was initially developed in *Ciona intestinalis* as this system provides very fast and efficient means to test within 10 hours the activity of proteins or cis-regulatory regions by electroporation. Our vector set is suitable to analyse the activity of the growing collection of publicly available cDNA clones sequenced from several attR1/R2 Gateway cDNA libraries. This collection currently covers 60% of the JGI version 1 gene set [41] and is being extended to generate a full ORF Unigene set in the frame of a 2006 JGI Community Sequencing project (http://www.jgi.doe.gov/sequencing/why/CSP2006/seasquirtcDNA.html). The electroporation vectors described in this article open the way to large scale gain-of-function screens by electroporation of small pools of cDNAs under control of a suitable driver, analogous to those successfully carried out in *Xenopus* and fish by the more tedious mRNA injection method. In addition, the Chordate vector suite allows to add either an N- or C-terminal Venus-YFP or CyanFP fluorescent protein to any gene product and thus to track the sub-cellular localisation of proteins of interest. To validate this strategy, we developed a first set of fluorescent reporters for different cellular organelles. The current collection comprises markers to track chromatin (HistoneH2B-venus), microtubules (Ensconsin), plasma membrane (GAP-43-GFP), and cytoplasm (Venus) and centrosomes (Aurora Kinase). These markers should help characterising at the cellular level the effect of gain or loss of gene function.

*Ciona intestinalis* is becoming a powerhouse for the characterisation of cis-regulatory sequences. It has a small compact genome, in which intergenic and intronic regions are much smaller than in vertebrates, and phylogenetic footprinting with the sequenced genome of *Ciona savignyi* was shown to be an efficient way to identify cis-regulatory modules. As a result, over 100 ascidian cis-regulatory sequences have been characterised over the past few years [42]. Using our novel attR3/R5Gateway cassette and its derivatives obtained by remobilising one DNA segment, one can test the activity of sets of full cis-regulatory sequences (L3/L5 cloning into a promoter-less vector), and putative enhancers (L3/L4 cloning in a vector containing a minimal promoter between the attB4 and attB5 sites). We validated the vectors on a first set of regulatory sequences which collectively drive expression throughout the ectoderm (pFOG), the endoderm (pCi-Titf1), muscle (pCi-Sna) and notochord (peBra) lineages of early embryos. These entry clones are part of the current vector suite release (See table page 24 of Manual S1). We are currently extending this collection by cloning candidate conserved regulatory sequences located in the vicinity of transcription factor genes.

### Towards a comparative functional genomics approach in chordates

*Ciona intestinalis* belongs to the tunicates, the closest living relatives of the vertebrates [4]. This phylogenetic position suggests that protein localisation and activity may be conserved between *Ciona* and vertebrates, a proposal strengthened by our demonstration that vertebrate Histone, GAP43 and ensconsin fusion proteins localise to the expected cellular compartment in *Ciona* cells. The compatibility of the Human ORFeome entry clones with the Chordate vector set is thus an encouragement to assay the subcellular localisation of uncharacterised human proteins in the simple embryonic context of *Ciona*. Such studies could be further extended to the *C. elegans* ORFeome, thus allowing to test the evolution of protein function across large evolutionary distances.

While it is expected that protein activity should be largely conserved within the chordates, the issue of the conservation of the activity of cis-regulatory sequences is open. In spite of sharing a common larval body plan, ascidians and vertebrates use very different embryological strategies, which could have led to a broad divergence in cis-regulation. Consistent with this, non-coding sequences conserved within vertebrates cannot be detected in the *Ciona* genome [43]. The existence of blocks of sequence conservation may however not be necessary for the conservation of enhancer activity [7]. It is at present difficult to estimate the level of conservation of regulatory logic between ascidians and vertebrates as there is a single report of a *Ciona* enhancer been introduced into vertebrate embryos and in this case the activity was not conserved [44]. Our demonstration that the chordate vector set is suitable for the analysis of *Ciona*, fish and mammalian enhancers is an incentive to generate collections of enhancers active in each organism and that can then be tested across the chordate phylum.

## Materials and Methods

### In Vitro Recombination Reactions

The BP reactions were performed in 10 µl with 50 femtomoles of attB-PCR product, and 50 femtomole of pDonor, 1 µl BP clonase mix (Invitrogen). LR reactions were performed in 10 µl using 10 femtomole of each plasmid and 1 µl of LR enzyme mix (Invitrogen) or LR–plus enzyme mix (multisite LR reactions, Invitrogen). Recombination reactions were incubated for 12h at room temperature and followed by Proteinase K treatment (10 min at 37°C) to inactivate the enzyme mix before transformation. Transformation of expression vectors was performed using 1–2 µl of recombination reaction and 40 µl of electrocompetent DH5α bacteria (efficiency equivalent or higher than 10^8^ cfu/µg for multisite Gateway reactions). Transformation of donor or destination vectors was done using Library Efficiency DB3.1 chemocompetent cells (Invitrogen).

### PCR amplification

All PCR amplifications to built pDest or pEntry clones were performed with high fidelity DNA polymerase (Pfx, Invitrogen). Flexi-taq (Promega) was used for routine clone screening.

### Origin of the animals

*Ciona* intestinalis were obtained from the Roscoff Marine Biology Station (Roscoff, France). Ascidian gamete collection, fertilisation and embryo cultures were as in [25]. *Zebrafish* embryos were collected from crosses of AB0 and Tübingen wild type strains kept under standard conditions.

### Electroporations

Plasmid DNA was isolated and purified using Nucleobond PC500 (Macherey Nagel).

The protocol was adapted from a previous report [25] except that all quantities were halved. Electroporation is performed in 0.4cm cuvettes with a BTX T820 (one pulse, 50V, 16 ms). Under these conditions, different cells of an embryo inherit different amounts of plasmid, leading to a mosaic expression of the transgene.

### Injection of mRNAs and plasmids

Microinjections of mRNA in ascidians were carried out as previously described [25]. All synthetic mRNA were transcribed with mMachine kit (Ambion). Approximately 30pl of solution at 20 ng/µl were injected per ascidian egg. Fertilized *zebrafish* eggs were injected manually at the one cell stage, with injection solutions containing 1% phenol red and the specified plasmids at 25 ng/µl. Injected embryos were kept at 28°C and collected for expression analysis at 30 h post fertilization. GFP constructs injected embryos were analysed live under an epifluorescence microscope. *lacZ* construct injected embryos were fixed and X-gal stained as described previously [45].

### Cell transfections

Cell transfections were carried out as previously described [31]. HaCat human keratinocytes and HeLa carcinoma cells were grown in Dulbecco's modified Eagle's medium–10% fetal bovine serum. Both cell types were transfected using Lipofectamine 2000 (Invitrogen), following the manufacturer's protocol. Cells were plated in 60mm dishes and transfected with 4þug of a Gateway expression construct containing the tk promoter and the various enhancer regions. To precisely compare the activity of pSP72-B3-CMV-B5::B1-NLS-LacZ-B2 and pCMV-LacZ (kind gift from R. Di Lauro), each of these construct was co-electroporated with and internal control, pCMV-EGFP (Clontech) used to normalize transfection efficiencies.

### X-Gal Staining and immunohistochemistry

X-gal staining was performed as previously described [25]. Anti-HA immunostaining was performed with a High Affinity Rat monoclonal antibody (1/200, Roche) incubated overnight at 4°C, followed by an Alexa 546 conjugated anti-rat antibody (1/500, Molecular Probe).

### Generation of novel pDONR vectors

attP3, attP5, attP4 and attP4R were derived from the attP1 and attP2 present in pDONR-221 by Overlap Extension PCR mutagenesis [46] using two perfectly matching primers outside of the attP, and two primers overlapping by 27 nucleotides and carrying point mutations in the region of the attPs that confer recombination specificity (see Table S1 for the sequence of the primers used).

To generate attP3 from attP1, this strategy leads to a 606bp fragment flanked by Apa1 and Pst1 restriction sites. This fragment is used to replace the corresponding attP1 fragment of pDONR-221. To generate attP4 and attP5 from attP2, the same strategy leads to a 721bp fragment flanked by EcoR1 and EcoRV, which are used to replace the corresponding attP2 fragment of pDONR-221. Replacement of Apa1-Pst1 flanked attP1 and EcoR1-EcoRV flanked attP2 sequences by their mutagenised counterparts in pDONR-221 led to pDonor attP3/P5, pDonor attP3/P4. PCR amplification of attP4 flanked with Apa1 and Pst1 was used to replace attP1 from pDONR-221-attP1/attP5 and gave rise to pDonor attP4R/P5. Note that the attP4R is a reverse oriented recombination site of the attP4, and gives rise to an attR4 site (instead an attL4) in pENTR clone. All theses pDONRs contain the ccdB and chloramphenicol resistance genes (Manual S1, page 24).

### Construction of the attR3-ccdB-CmR-attR5 cassette

The pFOG regulatory sequence [22] was PCR amplified with the pFOG attB3-fwd and pFOG-attB5-rev cloned into the EcoRV site of pBluescriptKS. This plasmid was BP recombined with an attP3-P5 cassette purified from Apa1 and EcoRV cut pDONR-221-attP3/P5. After recombination, the resulting pBS-attR3-ccdB-CmR-attR5 vector was selected on ampicillin and chloramphenicol LB agar DB3.1 bacteria. Positive clones were identified by restriction profile analysis and sequencing.

### Construction of the destination vectors

The injection vector pRN3 [27] was modified to give rise to pSPE3 by inserting a new polylinker EcoR1-Stu1-Pme1-EcoRV-Not1 (GAATTCAGGCCTTTGTTTAAACTTAGATATCGCGGCCGC) between the EcoR1 and Not1 sites. For this, the two primers pSPE3-Fw and pSPE3-Rev (Table S1) were annealed and cloned between EcoR1 and Not1.

The electroporation vector pSP72-1.27 [16] was modified to give rise to pSP72BSSPE by replacing the NLS-LacZ reporter gene, cloned between BamH1 and EcoR1 by a BamH1-Swa1-Stu1-Pme1-EcoRV-EcoR1 polylinker (GGATCCATTTAAATAGGCCTTTGTTTAAACTTAGATATCGGAATTC). For this, the two primers pSP72-Fw and pSP72-Rev (Table S1) were annealed and cloned between Swa1-EcoRV.

In both vectors, the *Reading Frame A* cassette (RfA), blunt cut by EcoRV was subsequently inserted into Pme1. The tag (Venus, CFP or HA) was amplified by PCR with high fidelity polymerase (*Pfx*, Invitrogen), gel purified and inserted by blunt restriction cloning into Stu1 (N-terminal tags) or EcoRV (C-terminal tags) (Primers and procedure detailed in Table S2 and Manual S1 page 4). In pSP72BSSPE-RfA vectors, cis-regulatory regions also amplified by PCR were inserted in Swa1 (Manual S1 page 5).

To generate the pSP72BSSPE-R3-R5::RfA or pSP72BSSPE-R3-R5::B1-ORF-B2 destination vector series, the attR3/R5 cassette was excised by Xho1-Xba1 digestion from pBS-attR3-ccdB-CmR-attR5 and cloned upstream of the RfA cassette into the same restrictions sites of pSP72BSSPE-RfA or pSP72BSSPE-B1-ORF-B2 vectors.

### Generation of ORF entry clones

The entry clones (pENTR) containing ORFs or cis-regulatory region were obtained by recombining an attB-flanked PCR product and the adequate pDONR vector. The primers used are presented in Table S3. A Kozak sequence or a stop codon were added to the the attB1 and attB2 primers when needed to generate fusion protein constructs as indicated in the Manual S1, page 11). Depending on individual cases, the attB-flanked PCR product was generated in one step using long oligonucleotides containing a region of overlap with the cDNA and the attBs, or in two steps using shorter overlapping primers. Because of the high frequency of the self-recombination of pDONR-221-attP4R/P5 resulting in the deletion of the ccdB and CmR genes, a PCR screen on pENTR-attR4/L5 with M13-fw/M13-rev primers was systematically applied to bacterial colonies.

ORFs entry clones were amplified (all primers described in Table S3) from clones previously described. GATAa: (Cieg021b07(AK114877), Ci-Aur : GC19c04 (Cieg023k05) both found in the Ci Gene Collection Release 1 [47]. NLS-lacZ and LacZ : [16]. TauLacZ: [48]. Histone 2B: [49]. Mouse Gap-43-GFP: [19], [20]. Venus: [50], Ensconsin-3GFP: [51].

### Generation of cis-regulatory region entry clones

The pFOG and pTitf cis-regulatory regions, FOG basal promoter, eBra and pSna enhancers, were PCR amplified from genomic DNA using the primers indicated iinn in Table S3.

The viral CMV regulatory region, including enhancer and promoter, was amplified from pCMV-LacZ using attB3CMV-1 (ATAAAGTAGGCTAGGCCCTTTCGTCTTCACTC) and attB5CMV-1 (CAAAAGTTGGGTTTAAAAAGTGTTCGAGGGGAAA). The mammalian enhancer regions C15, C40 and C50 were amplified from previously generated tk-Luc containing plasmids [31], using attB3EnhTK (ATAAAGTAGGCTTAGGCTGTCCCCAGTGCAAG) and attB5EnhTK (CAAAAGTTGGGTAACAGTACCGGAATGCCAAGC) designed on the plasmid and tk promoter sequence respectively. Purified PCR products were further amplified with attB3adapt (GGGGACAAGTTTGTATAATAAAGTAGGCT) and attB5adapt (GGGGACCACTTTGTATACAAAAGTTGGGT), and recombined into pDONR-221-P3/P5 using BP clonase to obtain the pENTR clones. The pENTR clones containing the enhancer-tk sequences were recombined with pSP72BSSPE-R3-ccdB/CmR-R5::B1-NLS-lacZ-B2.

The *zebrafish* shh 2.4kb cis-regulatory region was amplified from the pCS2+-2.4shh:GFP vector with the shh2.4FP-B3 (ATAAAGTAGGCT-GTCGACTATTTACTATTTTTT TAGTG) and shh2.4RP-B5 (ATAAAGTAGGCT-CTCGAGCGGTCTGTCTAGCAG) primers. The second round of PCR was performed with attB3adapt and attB5adapt primers. The purified PCR product was BP recombined into pDONR-221-P3/P5. The generated entry clone was sequenced and LR recombined into pSP72BSSPE-R3-ccdB/CmR-R5::B1-NLS-lacZ-B2, or co-recombined with pENTR-attL1-Kozak-Venus-Stop-attL2 into pSP72BSSPE-R3-ccdB/CmR-R5::RfA.

## Supporting Information

Manual S1 Chordate Gateway Vector Manual
(0.42 MB PDF)

Table S1 (0.06 MB DOC)

Table S2 (0.05 MB DOC)

Table S3 (0.09 MB DOC)
