# Supplementary material for: A Multicassette Gateway Vector Set for High Throughput and Comparative Analyses in Ciona and Vertebrate Embryos
Source: PLoS One. 2007 Sep 19;2(9):e916. doi: 10.1371/journal.pone.0000916 (PMC1976267; doi:10.1371/journal.pone.0000916)
Supplement: Manual S1 — Chordate Gateway Vector Manual (0.42 MB PDF) [file pone.0000916.s001.pdf]

GATEWAY VECTORS FOR IN VIVO ANALYSIS OF REGULATORY REGIONS, GENE FUNCTION, AND CELLULAR LOCALISATION

Agnès Roure, Ute Rothbächer and Patrick Lemaire

May 2007 release

Version 6.7

(including location of clones in distributed plate)

Contacts: roure@ibdml.univ-mrs.fr, lemaire@ibdml.univ-mrs.fr

Table of contents

Page numbers

Principle of the Gateway system **2**

I) General presentation of strategy used **3**

- General strategy: using an ORF entry clone to produce expression clones for mRNA synthesis 4
- General strategy: using an ORF entry clone to generate transgenesis clones 5
- Tags available in destination vectors 6
- Naming conventions for donor, entry, destination and expression vectors 7

II) Design of ORF entry clones **8**

- Sequence around the ORF in the expression clones 9
- Design of ORF entry clones: PCR amplification of the ORF 10
- Primer sequences for tagged, untagged ORF overexpression 11
- Available entry clones to track cells and organelles 12

III) Design of entry clones for cis-regulatory sequences **13**

- Design of cis-regulatory entry clones : available donor vectors 14
- Design of cis-regulatory entry clones : PCR amplification of the cis-regulation sequence 15
- Cis-regulatory primer sequences 16
- Available cis-regulatory entry clone 17

IV) Generation of complex transgenesis constructs from a collection of destination vector and entry clones **18**

- Available transgenesis vector with 2 Gateway cassettes 19
- Inserting simultaneously an ORF of interest and a cis-regulatory region in a 2-cassette transgenesis destination vector 20
- Removing a cis-regulatory region or an ORF from a complex transgenesis expression vector 21
- Inserting a new cis-regulatory region or ORF in a Gateway destination vector 22
- Cloning of an enhancer and minimal promoter in front of an ORF of interest 23

V) Position of vectors available in the distributed 96 wells plate **24**

Principle of the Gateway system

<u>Aim:</u> This system, commercially developed by Invitrogen, was designed to avoid the difficulties associated with restriction enzyme mediated cloning. It makes use of phage Lambda recombinases (Landy, 1989) to recombine efficiently in vitro PCR amplified molecules and vectors (see Invitrogen Gateway manual: http://www.invitrogen.com/content/sfs/manuals/getwayman.pdf). The basic principle is to generate, in a first reaction, « shuttle » vectors (the Entry clones) containing the molecule of interest. These are then recombined in a variety of Destination vectors to generate the expression clones of interest. The enzymes involved in the 2 reactions differ. The power of the system lies in the positive (Chloramphenicol resitance) and negative (ccdB) selection of genes found in the Gateway cassette (in purple), and on the use of different antibiotic resistance genes in Donor and Destination vectors.

Ref: Landy A. Dynamic, structural, and regulatory aspects of lambda site-specific recombination. Annu Rev Biochem. 1989; 58:913-49.

<u>1- BP reaction</u>

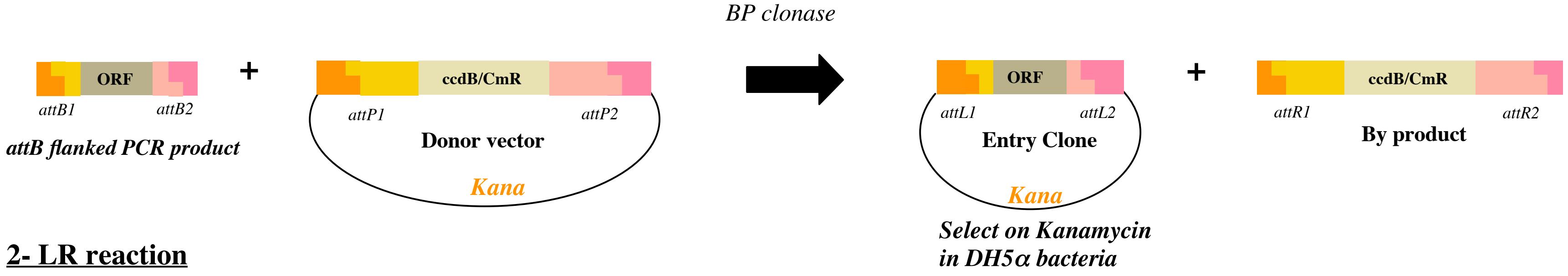

<u>2- LR reaction</u>

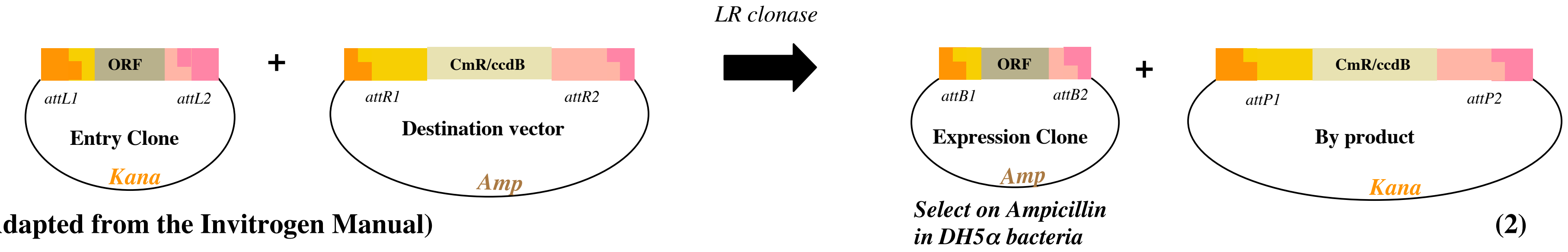

(Adapted from the Invitrogen Manual)

(2)

I) General presentation of the strategy used

Aim: This section presents the general strategy we used to adapt the Gateway system to developmental biology purposes. We wanted to have a vector suite in which the same ORF entry clones could be used in vectors aiming at making synthetic mRNA for injection and for transgenesis. We also wanted a flexible system in which different Gateway cassettes could be used in transgenesis vectors to clones ORFs and Cis-regulatory sequences. The following slides first display the architecture of the destination vectors we generated, and the available tags. A last slide presents the naming convention for all vectors.

General strategy: using an ORF entry clone to produce expression clones for mRNA synthesis.

<u>Aim:</u> The same ORF entry clones are used in two types of vectors either to make synthetic mRNA, or for transgenesis. The mRNA destination/expression clones are composed as shown below. The ORF is flanked by the recombination sequences attB1 and B2 with or without flanking Tags (see p6 for available tags).

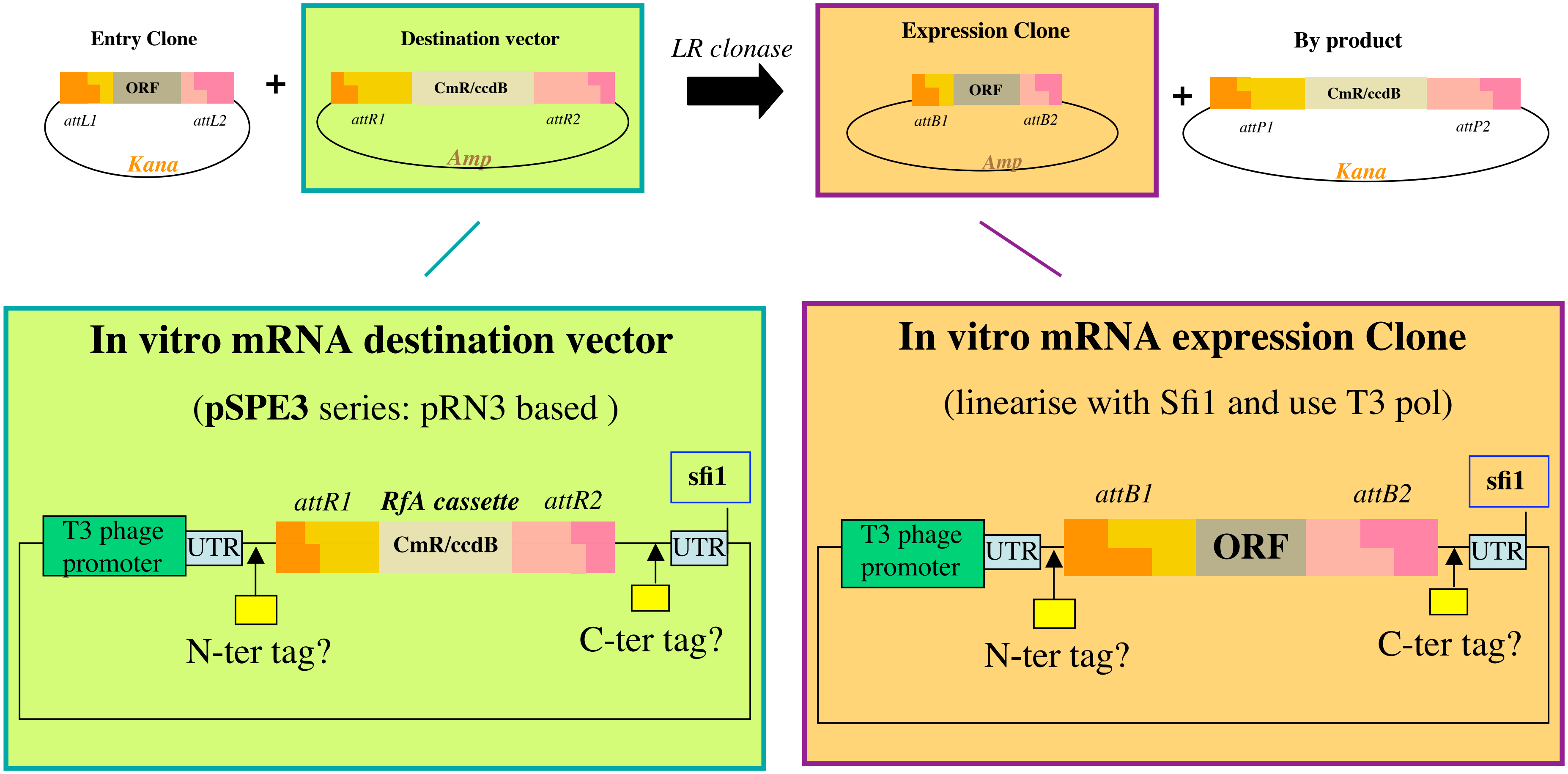

General strategy: using an ORF entry clone to generate transgenesis clones.

Aim: The same ORF entry clones are used in two types of vectors either to make synthetic mRNA (previous page), or for transgenesis. The resulting destination/expression clones for transgenesis should look as shown here. The ORF is flanked by the recombination sequences attB1 and B2 with or without flanking Tags (see p6 for available tags). The cis-regulatory driver can be either cloned in a Swa1 site or Gateway cloned using different recombination feet (attB3/B5) at the same time as the ORF (see p19).

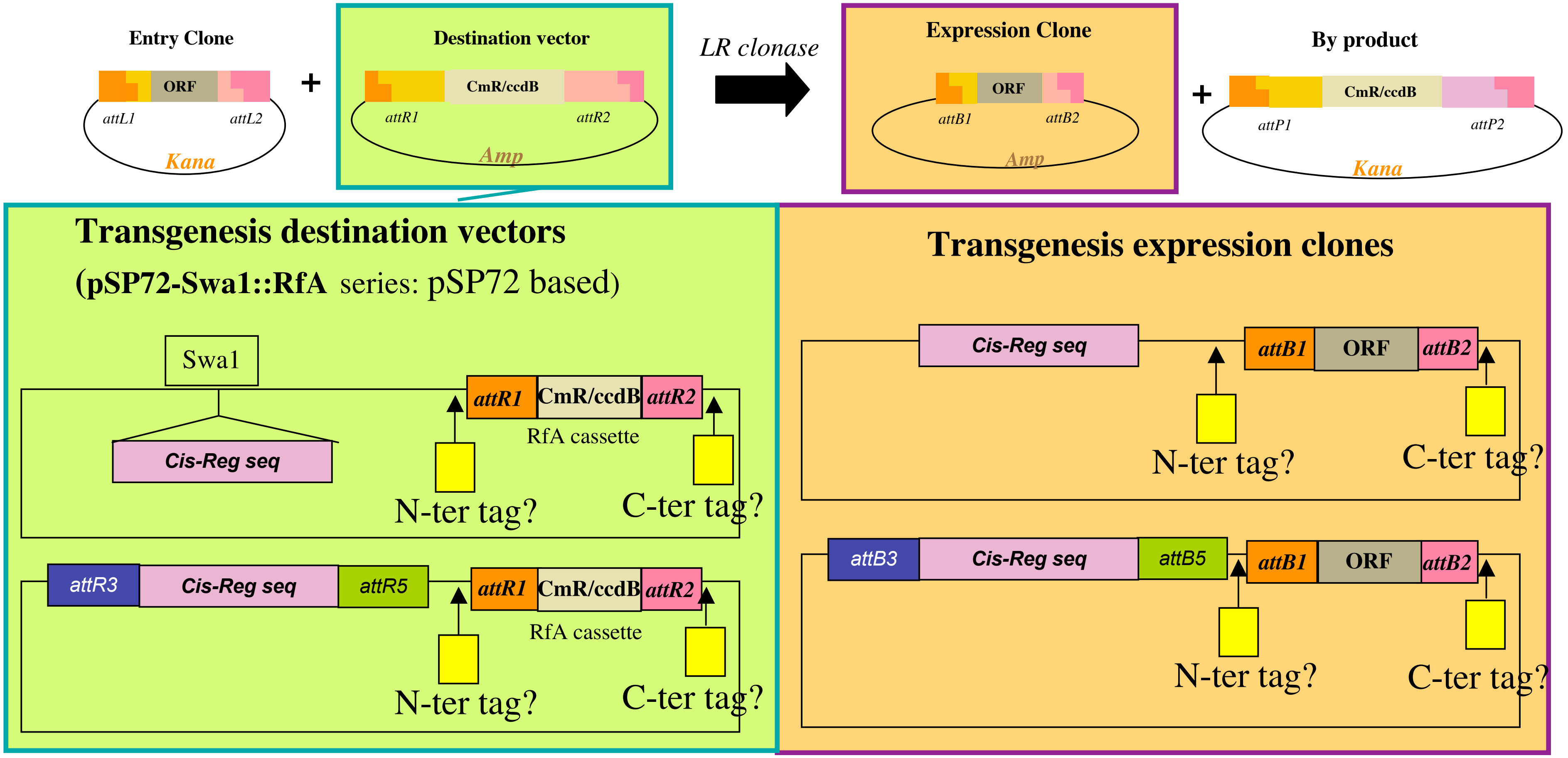

Tags available in destination vectors (June 2006 release)

Aim: This schema presents the various configurations of destination vectors for ORF cloning available for either synthtic mRNA production or transgenesis.

IMPORTANT: the cassette for ORF insertion **is ALWAYS RfA** (*Reading frame A*) using attR1/R2 feet

mRNA synthesis pDest

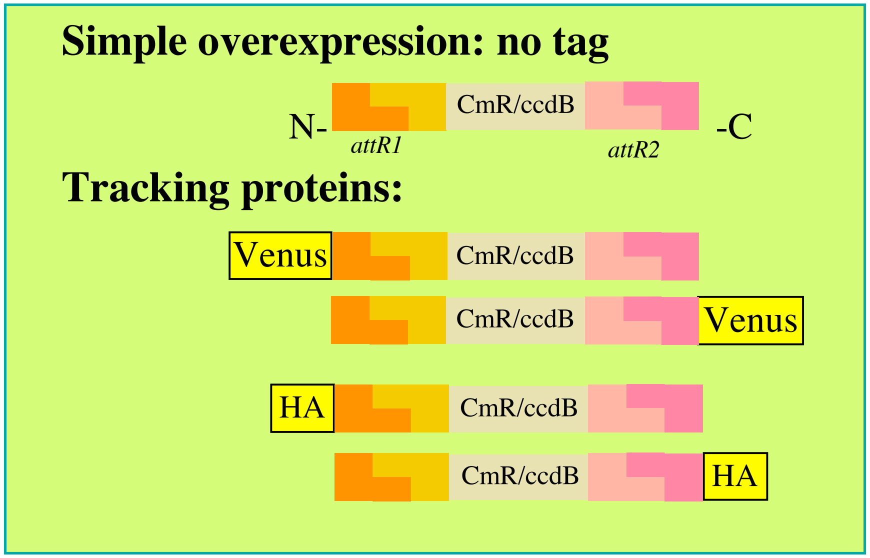

Transgenesis pDest

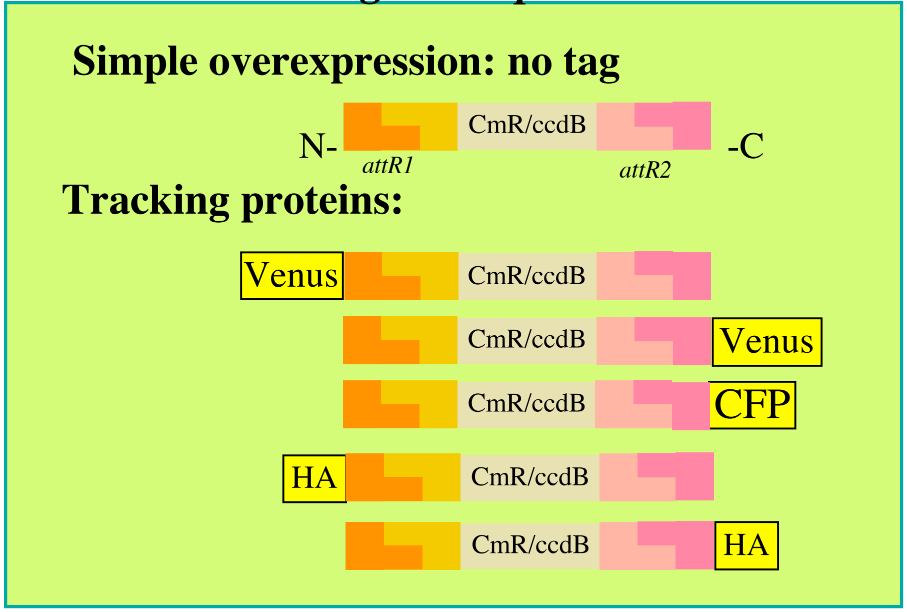

Naming conventions for donor, entry, destination and expression vectors

Where R1-A-R2 indicates an RfA cassette with attR1 and attR2 feet. R1-A-R2 is always used for ORFs, R3-ccdB/cmR-R5 for cis-reg regions.

Donor vectors :
« pDONR-221-Px-Py » where the Ps indicate the type of attPs used. Ex : pDONR-221-P1-P2
pDONR-221-P4R-P5

Entry clones :
« pENTR-L1-ORFname-L2 » where the Ls indicate the type of attLs used. Ex: pENTR-L1-GATAa-L2
« pENTR-L3-cis-regulatory region-L5 » pENTR-L3-pFOG-L5
« pENTR-L3-enhancer-L4 » pENTR-L3-a element Otx-L4
« pENTR-R4-minimal promotor-L5 » pENTR-R4-bpFOG-L5

Destination vectors mRNA production :
« pSPE3-R1-A-R2-Tag » for vectors with C-terminal tag Ex: pSPE3-R1-A-R2-Venus
« pSPE3-tag-R1-A-R2 » for vectors with N-terminal tag pSPE3-HA-R1-A-R2

Destination vectors transgenesis : (the :: symbol separates the driver or driver cassette from the expression cassette)
« pSP72-cis reg-min prom::R1-A-R2-Tag » Ex: pSP72-pFOG::R1-A-R2
« pSP72-R3-ccdB/cmR-R5::R1-A-R2» 2 cassettes, no tag pSP72-R3-ccdB/cmR-R5::R1-A-R2
« pSP72-R3-ccdB/cmR-R5::R1-A-R2-tag » 2 cassettes, C-ter Tag pSP72-R3-ccdB/cmR-R5::R1-A-R2-Venus
« pSP72-R3-ccdB/cmR-R5::tag-R1-A-R2 », 2 cassettes, N-ter Tag pSP72-R3-ccdB/cmR-R5 ::Venus-R1-A-R2

Expression clones : (the :: symbol separates the driver from the ORF)
« pRN3-B1-ORFname-B2-Tag » for RNA vectors with C-terminal tag Ex : pRN3-B1-GATAa-R2-Venus
« pSP72-cis reg-prom min::B1-ORFname-B2-tag » pSP72-pFOG::B1-GATAa-B2-CFP
« pSP72-B3-cis-reg-B5::tag-B1-ORFname-B2 » pSP72-B3-pFOG-B5::Venus-B1-GATAa-B2
« pSP72-B3-cis-reg-B5::B1-ORFname-B2-tag » pSP72-B3-pFOG-B5::B1-GATAa-B2-HA

II) Design of ORF Entry clones

Aim: This section presents the final sequence of the ORF portion of expression clones after recombination of one of the previous destination vectors with an ORF entry clone. It then illustrates how an ORF PCR fragment is generated to recombine into the donor vectors to create entry clones. Finally, it presents some available entry clones to track cells and sub cellular structures. The exact same strategy is used for RNA and transgenesis vectors.

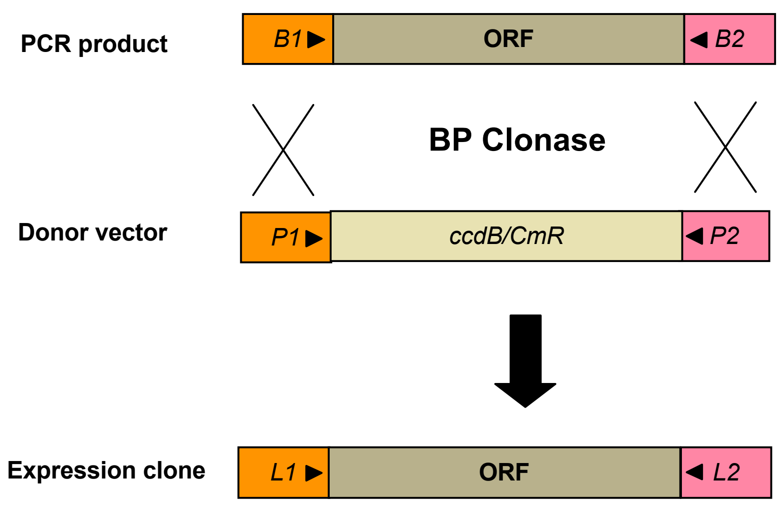

All entry clones should be selected on Kanamycin in DH5α bacteria

Sequence around the ORF in the expression clones

Aim:
The sequence around the ORF in the different types of expression clones is determined by the starting sequence of the PCR product to make the entry clones. Understanding this slide is therefore required to be able to design suitable entry clones. The tag used in the example is the HA tag. You should respect the reading frames in the same way for the other tags.

No Tag :
5'ATCACAAGTTTGTACAAAAAAGCAGGCTCAGAAAAAATG...TAAACCCAGCTTTCTTGTACAAAGTGGTGAT 3'

N terminal tag :
5'CAGAAAAAATG TAC CCA TAC GAC GTG CCA GAC TAC GCT CCT TTG TTT ATC ACA AGT TTG TAC AAA AAA GCA GGC TTG ATG…TAA ACCCAGCTTTCTTGTACAAAGTGGTGAT 3'

C-terminal tag :
5'ATCACAAGTTTGTACAAAAAAGCAGGCTCAGAAAAA ATG…AAC CCA GCT TTC TTG TAC AAA GTG GTG ATA AAC TTA GAT ATG TAC CCA TAC GAC GTG CCA GAC TAC GCT TGA 3'

HA tag, initiator/stop codon underlined
attB1; attB2
kozak sequence
initiator and stop codon of ORF
intervening polylinker sequences used to insert the tags.
extra-nucleotide added to keep the frame between the ORF and the tag

Design of ORF entry clones: PCR amplification of the ORF

<u>Aim:</u>
To generate an entry clone, one amplifies the ORF to flank it with the relevant attB sequences and to introduce a Kozak/ATG and stop codon for untagged expression or either one for in frame tagging. If a single PCR is used, the PCR primers would be very long and may contain mutations in a significant number of cases. The PCR is therefore done in two steps. The first step primers match the ORF and include only part of the attBs. The second step primers include the whole attBs, are generic and the same for all ORFs and tagging strategies. The sequence of the PCR primers can be found overleaf.

Part of attL1 *Part of attL2*

No Tag :
Final entry clone: 5′-TATAATGCCAACTTTGTACAAAAAAGCAGGCTCAGAAAAAATG…ORF…TAAACCCAGCTTTCTTGTACAAAGTTGGCATTA-3′
First PCR product : 5′-AAAAAGCAGGCTCAGAAAAAATG…ORF…TAAACCCAGCTTTCT-3′
Second PCR product : 5′-GGGGACAAGTTTGTACAAAAAAGCAGGCTCAGAAAAAATG…ORF…TAAACCCAGCTTTCTTGTACAAAGTGGTCCCC-3′

N terminal tag :
Final entry clone : 5′-TATAATCACAACTTTGTACAAAAAAGCAGGCTTGATG…ORF…TAAACCCAGCTTTCTTGTACAAAGTTGGCATTA 3′
First PCR product: 5′-AAAAGCAGGCTTGATG…ORF…TAAACCCAGCTTTCT-3′
Second PCR product: 5′-GGGGACAAGTTTGTACAAAAAAGCAGGCTTGATG…ORF…TAAACCCAGCTTTCTTGTACAAAGTGGTCCCC-3′

C-terminal tag :
Final entry clone : 5′-TATAATCACAACTTTGTACAAAAAAGCAGGCTCAGAAAAAATG…ORF…AACCCAGCTTTCTTGTACAAAGTTGGCATTA-3′
FirstPCR product: 5′-AAAAAGCAGGCTCAGAAAAAATG…ORF…AACCCAGCTTTCT-3′
Second PCR product : 5′-GGGGACAAGTTTGTACAAAAAAGCAGGCTCAGAAAAAATG…ORF…AACCCAGCTTTCTTGTACAAAGTGGTCCCC-3′

attB1, attB2, kozak sequence
initiator and stop codon of ORF
pDONR-221 sequence
extra-nucleotide added to keep the frame between the ORF and the tag

Primer Sequences for overexpression of tagged or untagged ORFs

Aim:

attB1and attB2 primer sequences to amplify an ORF to generate entry clones. These entry clones will be recombined in different pDest giving rise to 3 type of expression clones : untagged ORF (A), fusion protein with a tag in N-ter (B) or in C-ter (C).

•Choose the length of the primer sequence overlapping the ORF to reach a Tm of 60-65°C (Tm= 4x(G+C)+2x(A+T).

•The full length of your primer (including attB sequence) will then have a Tm much higher.

•The Kozak sequence used is in blue, included in the primer

•Stop codon sequence is in grey « TTA » in attB2 primer

A) Sequences for overexpression : no tag

attB1 fw = 5'-GGGGACAAGTTTGTACAAAAAAGCAGGCTCAGAAAAA-ATG-ORF-3'
attB1 adaptor = 5'-GGGGACAAGTTTGTACAAAAAAGCAGGCT
attB1-template primer = 5'-AAAAAGCAGGCTCAGAAAAA-ATG-ORF

attB2 rev = 5'-GGGGACCACTTTGTACAAGAAAGCTGGGT-TTA-ORF-3'
attB2-adaptor = 5'-GGGGACCACTTTGTACAAGAAAGCTGGGT
attB2-template primer = 5'-AGAAAGCTGGGT-TTA-ORF

B) Sequences for fusion protein : tag in N-ter

attB1 fw = 5'-GGGGACAAGTTTGTACAAAAAAGCAGGCT**TG**-ATG-ORF-3'
attB1 adaptor = 5'-GGGGACAAGTTTGTACAAAAAAGCAGGCT**TG**
attB1-template primer = 5'-AAAAAGCAGGCT**TG**-ATG-ORF

attB2 rev = 5'-GGGGACCACTTTGTACAAGAAAGCTGGGT-TTA-ORF
attB2-adaptor = 5'-GGGGACCACTTTGTACAAGAAAGCTGGGT
attB2-template primer = 5'-AGAAAGCTGGGT-TTA-ORF

C) Sequences for fusion protein : tag in C-ter

attB1 fw = 5'-GGGGACAAGTTTGTACAAAAAAGCAGGCTCAGAAAAA-ATG-ORF-3'
attB1 adaptor = 5'-GGGGACAAGTTTGTACAAAAAAGCAGGCT
attB1-template primer = 5'-AAAAAGCAGGCTCAGAAAAA-ATG-ORF

attB2 rev = 5'-GGGGACCACTTTGTACAAGAAAGCTGGGT**T**-ORF
attB2-adaptor = 5'-GGGGACCACTTTGTACAAGAAAGCTGGGT**T**
attB2-template primer = 5'-AGAAAGCTGGGT**T**-ORF

Available Entry clones to track cells and organelles

Aim: To help track cells and organelles, we generated a first series of entry clones to overexpress fluorescent or LacZ fusion proteins in different cell compartments. All clones were made by recombining an ORF PCR product into pDONR-221-P1-P2

Membrane :	pENTR-L1-Kozak-GFP-GPI-stop-L2 (Hiscoxa et al.2002)
	pENTR-L1-Kozak-GAP43-GFP-stop-L2 (Miriyoshi et al. 1996, Kim et al.1998)
Baso-lateral membranes :	pENTRY-L1-Dm-E-Cadherin-L2 (*, Oda et al., 2001)
Nucleus :	pENTR-L1-Kozak-HistH2B-L2 (*, Koster et al., 2001)
	pENTR-L1-Kozak-NLS-LacZ-stop-L2 (Corbo et al.,1997)
Microtubules:	pENTR-L1-Kozak-tau-LacZ-stop-L2 (Monbaerts et al., 1987)
	pENTR-L1-Kozak-Ensconsin-3xGFP-stop-L2 (Lenart, 2005)
Centrosome :	pENTR-L1-Kozak-AuroraKinase-L2 (*, F. Robin unpublished)
Cytoplasm :	pENTR-L1-Kozak-lacZ-stop-L2 (Corbo et al, 1997)
	pENTR-L1-Kozak-venus-stop-L2 (Nagai et al. 2002)

All entry clones should be recombined in an untagged R1-A-R2 destination cassette except (*) which should be recombined into a destination vector with C-ter fluorescent protein tag. This ORF therefore has no stop codon in its 3' end.

III) Design of Entry clones for cis regulatory sequences

Aim: Unlike the ORF clones, the frame of the cis regulatory sequences is not important. This section first presents the three donor vectors that can be used to clone cis-reg sequences, enhancers and minimal promoters. It then illustrates how the Cis reg PCR fragment is generated to recombine into the donor vectors to create entry clones. Finally, it presents some available cis-regulatory Entry clones included in the release.

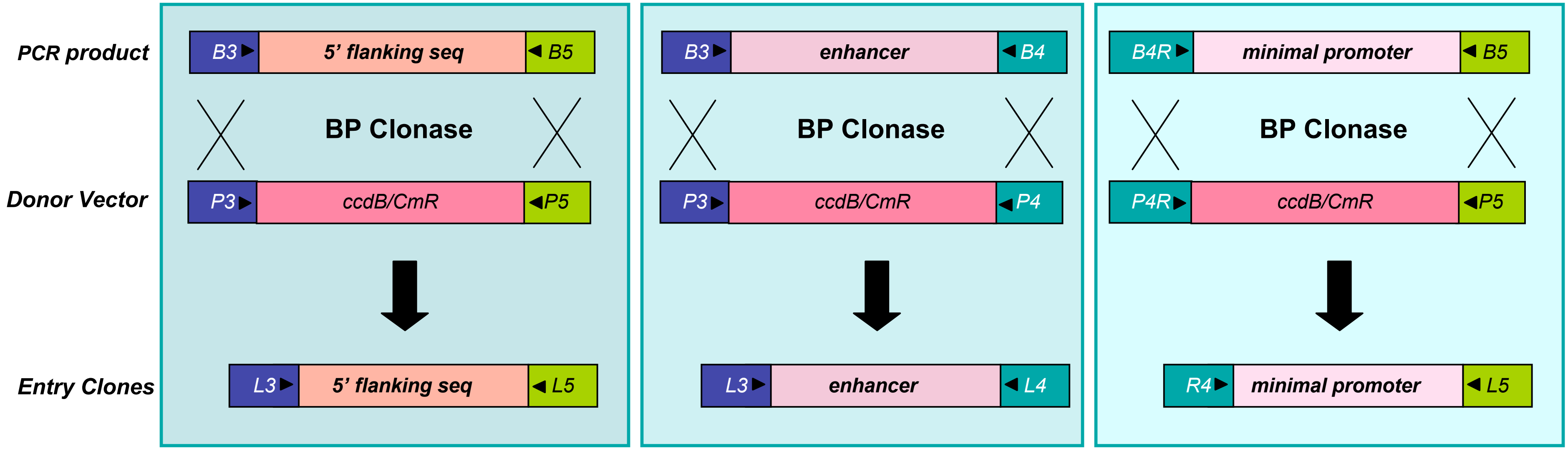

Example of the generation of an entry clone for a 5' flanking region, enhancer and minimal promoter. Different donor vectors allow to generate entry clones for enhancers or minimal promoters (see following pages).
The entry clones should be selected on Kanamycin in DH5α bacteria

Design of Cis-Reg entry clones: available donor vectors

Aim: To generate cis-regulatory entry clones that recombine in the feet of the cis-reg cassette of transgenesis destination vectors, we modified the pDONR-221 donor vector to exchange the attP1 and attP2 feet with a attP3, attP4, attP4R, or attP5 feet. Available donor vectors:

pDONR 221 (also called pDONR 221 P1-P2): to clone ORFs
pDONR 221 P3-P5: to clone 5' flanking regions
pDONR 221 P3-P4: to clone enhancers
pDONR 221 P4R-P5: to clone minimal promoters.

This slide indicates the sequence of the core portion of the resulting feet.

Comparison of attP1 with attP3 and attP4R

```
attP4R           5'  CAAC TTT TCT ATA CAA AGT TG   3'
attP3            5'  CAAC TTT GTA TAA TAA AGT TG   3'
attP1     5' TAA TGC CAAC TTT GTA CAA AAA AGC TGA ACG AGA  3'
attP1     3' ATT TCG GTTG AAA CAT GTT TTT TCG ACT TGC TCT  5'
attP3            3'  GTTG AAA CAT ATT ATT TCA AC   5'
attP4R           3'  GTTG AAA AGA TAT GTT TCA AC   5'
```

upper strand (attP4R, attP3, attP1 5'→3')

lower strand (attP1, attP3, attP4R 3'→5')

Comparison of attP2 with attP4 and attP5

```
attP4             5'  CA ACT TTT CTA TAC AAA GTT G  3'
attP5             5'  CA ACT TTT GTA TAC AAA GTT G  3'
attP2     5' TCT CGT TCA GCT TTC TTG TAC AAA GTT GGC ATT A  3'
attP2     3' AGA GCA AGT CGA AAG AAC ATG TTT CAA CCG TAA T  5'
attP5             3'  GT TGA AAA CAT ATG TTT CAA C  5'
attP4             3'  GT TGA AAA GAT ATG TTT CAA C  5'
```

upper strand (attP4, attP5, attP2 5'→3')

lower strand (attP2, attP5, attP4 3'→5')

Design of Cis-Reg entry clones: PCR amplification of the Cis-reg sequence

Aim:

In contrast to ORF entry clones, the phase is not important in the design of Cis-reg entry clones. The same two step PCR strategy is used to reduce the length of the PCR primers used. The slide shows how to design the PCR primers to obtain an entry clone of a given sequence. Note that the outside feet of the 2nd PCR and entry clone differ as a result of the recombination process. The precise PCR primers used to amplify Cis-reg regions can be found overleaf.

Cloning a Cis-regulatory region in a P3/P5 donor vector

```
                                 Part of attL3                              Part of attL5
Final entry clone: 5'…TTTTTTATAATGCCAACTTTGTATAATAAAGTAGGCT….cis-reg... ACCCAACTTTTGTATACAAAGTTGGCATTATAAGAAA …3'
First PCR product:                         5'-ATAAAGTAGGCT….cis-reg... ACCCAACTTTTG- 3'
Second PCR product:         5'-GGGGACAAGTTTGTATAATAAAGTAGGCT….cis-reg... ACCCAACTTTTGTATACAAAGTGGTCCCC- 3'
```

Cloning an enhancer in a P3/P4 donor vector

```
                                 Part of attL3                              Part of attL4
Final Entry clone: 5'…TTTTTTATAATGCCAACTTTGTATAATAAAGTAGGCT….cis-reg... ACCCAACTTTTCTATACAAAGTTGGCATTATAAGAAA…3'
1st PCR product:                           5'-ATAAAGTAGGCT….cis-reg... ACCCAACTTTTC- 3'
2nd PCR product:            5'-GGGGACAAGTTTGTATAATAAAGTAGGCT….cis-reg... ACCCAACTTTTCTATACAAAGTGGTCCCC- 3'
```

Cloning a minimal promoter in a P4R/P5 donor vector

```
                                 Part of attR4                             Part of attL5
Final Entry clone:  5'…ACGTTTCTCGTTCAACTTTTCTATACAAAGTGGCA….cis-reg... ACCCAACTTTTGTATACAAAGTTGGCATTATAAGAAA …3'
1st PCR product:                           5'-ACAAAGTGGCA….cis-reg... ACCCAACTTTTG- 3'
2nd PCR product:            5'-GGGGCCAAGTTTTCTATACAAAGTGGCA….cis-reg... ACCCAACTTTTGTATACAAAGTGGTCCCC- 3'
```

Cis-regulatory primer sequences

Choose the length of the part of overlapping the cis-regulatory region to reach a Tm= 60-65°C. The whole primer Tm, including attB sequence will be much higher.

Underlined sequences correspond to the recombination sites

```
attB3 fw :                      5'-GGGGACAAGTTTGTATAATAAAGTAGGCT-cis regulatory
attB3-adaptor :                 5'-GGGGACAAGTTTGTATAATAAAGTAGGCT
S-attB3-template primer :                        5'-ATAAAGTAGGCT-cis regulatory

attB4 rev :                     5'-GGGGACCACTTTGTATAGAAAAGTTGGGT-cis regulatory
attB4-adaptor-rev :             5'-GGGGACCACTTTGTATAGAAAAGTTGGGT
S-attB4-template primer:                         5'-GAAAAGTTGGGT-cis regulatory

attB4R fw :                     5'-GGGGCCAAGTTTTCTATACAAAGTGGCA-cis regulatory
attB4R-adaptor-fw:              5'-GGGGCCAAGTTTTCTATACAAAGTGGCA
S-attB4R-template primer :                       5'-ACAAAGTGGCA-cis regulatory

attB5-rev :                     5'-GGGGACCACTTTGTATACAAAAGTTGGGT-cis regulatory
attB5-adaptor-rev :             5'-GGGGACCACTTTGTATACAAAAGTTGGGT
S-attB5-template primer :                        5'-CAAAAGTTGGGT-cis regulatory
```

Available Cis-regulatory Entry clones

At the present only a small number of entry clone are released. The collection will be enhanced with time provided you do not forget to contribute your own clones to the collection.

• *attL3-pFOG-attL5*	Ci-FOG cis-regulatory region, drives expression in animal territory from 16 cell stage, (Pasini et al., 2006; Lamy et al., 2006)
• *attL3-pSnail-attL5*	Ci-Snail cis-regulatory region drives expression in mesodermal cells, (Erives et al.1998)
• *attL3-a element Otx-attL4*	Otx cis-regulatory region, drives expression in a6.5, b6.5 lineages from late 32-cell stage, (Bertrand et al., 2003)
• *attL3-eBra-attL4*	Brachury cis-regulatory region, drives expression in notochord lineage (Corbo et al., 1997)
• *attL3-Titf1-attL4*	Titf1 cis-regulatory region, drives expression in endoderm ligneage (Fanelli et al., 2003)
• *attR4-bpFOG-attL5*	Strong basal promoter of FOG, (U.R. and P.L. , unpublished)

IV) Generation of complex transgenesis constructs from a collection of destination vectors and Entry clones.

Aim: The previous sections presented the destination vectors and Entry clone design. This section shows how to use these vectors to generate complex transgenesis constructs. It shows how to simultaneously introduce a Cis-reg and an ORF entry clones into a 2-cassette destination vector, and how to selectively exchange either the Cis-reg sequence or the ORF. It finally shows how an enhancer and a minimal promoter can simultaneously be introduced into a destination vector.

Available transgenesis destination vectors with two Gateway cassettes

The release includes two types of 2 cassettes destination vectors. One type is ready to accept 2 entry clones simultaneously. In the other type of vectors one cassette has already been used to insert either a cis-regulatory sequence or an ORF, the other remaining free.

<u>**Aim**</u> **:** Insert1 or 2 entry clones (L1/L2 and L3/L5) in two different gateway casettes (R3/R5 and R1-A-R2) or insert 1 entry clone (L3/L5) in a pDest-containing attB1-ORF-attB2

Destination vectors for the recombination of 2 entry clones.

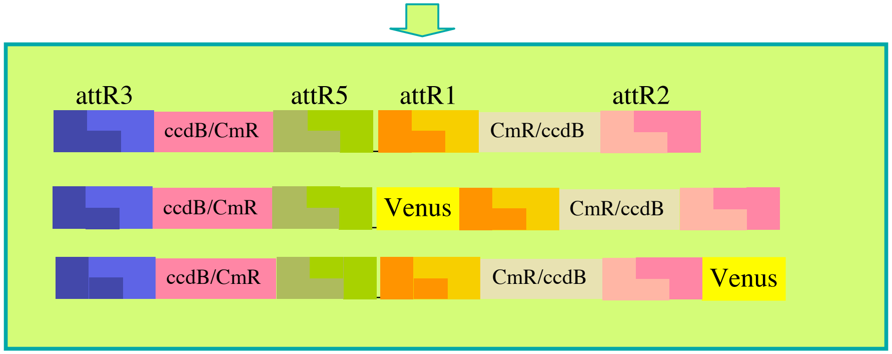

Destination vectors for the recombination of 1 entry clone

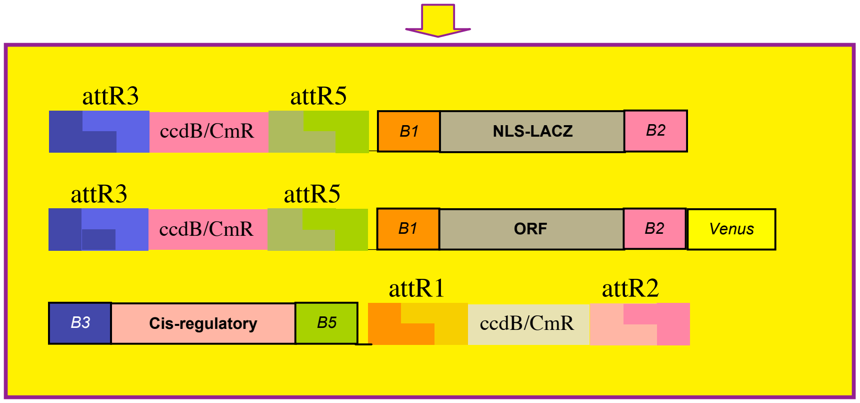

Inserting simultaneously an ORF of interest and a cis-regulatory region in a 2-cassette transgenesis destination vector.

<u>Aim:</u>
Starting from two PCR products flanked by attBs, one makes two entry clones, one with a Cis-reg region using the P3/P5 feet, the other with an ORF of interest, using the P1/P2 feet. These are simultaneously recombined in a one-tube reaction in a pDest vector to generate an expression vector in which the ORF, tagged or untagged is under control of the cis-reg region.
Selection of the recombinants is done on Ampicillin in DH5α bacteria

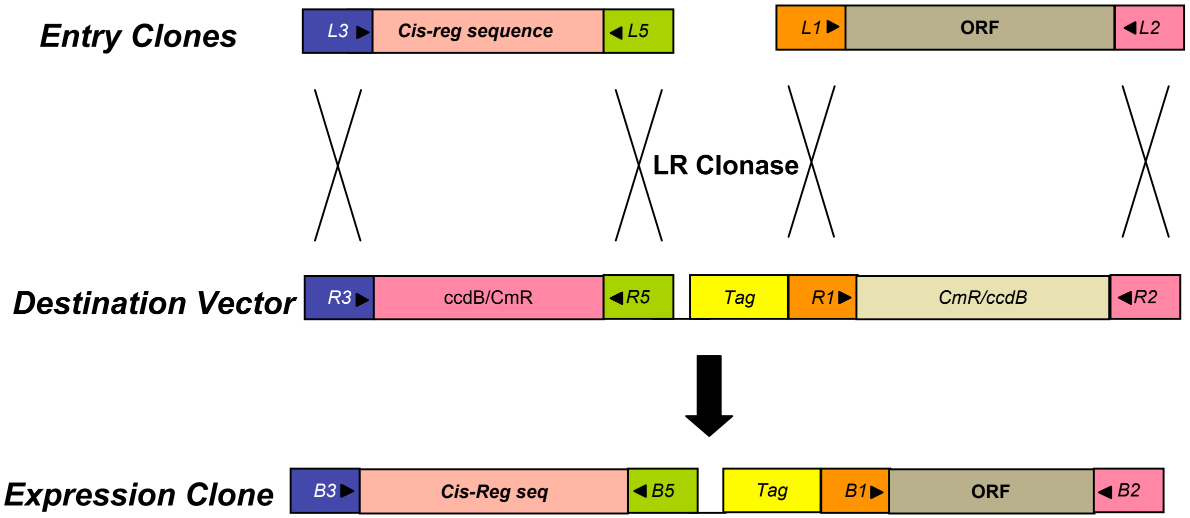

<u>What you need:</u>
1. pENTR-L3-cis reg-L5 entry clone
2. pENTR-L1-ORF-L2 entry clone
3. A pSP72-R3-ccdB/cmR-R5::R1-A-R2 with or without tag (here an N-ter Tag is shown, but the vectors exist with N-ter C-ter tag or without tag (see p19).

Removing a cis-regulatory region or an ORF of interest from a complex Gateway expression vector.

Aim:
This is the first step of the exchange of either the Cis-reg or the ORF. You will end up with a destination vector in which a single cassette can be used. The final destination vector is selected on ampicillin plus chloramphenicol in DB3.1 bacteria

Cis-reg removal:

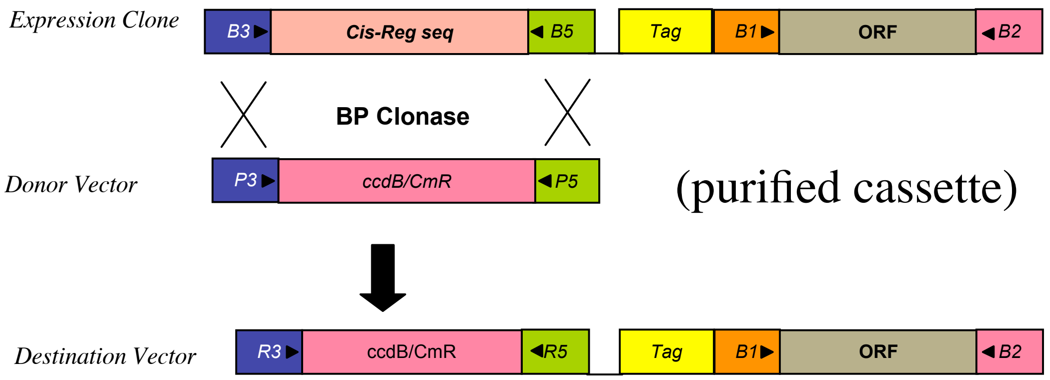

ORF removal:

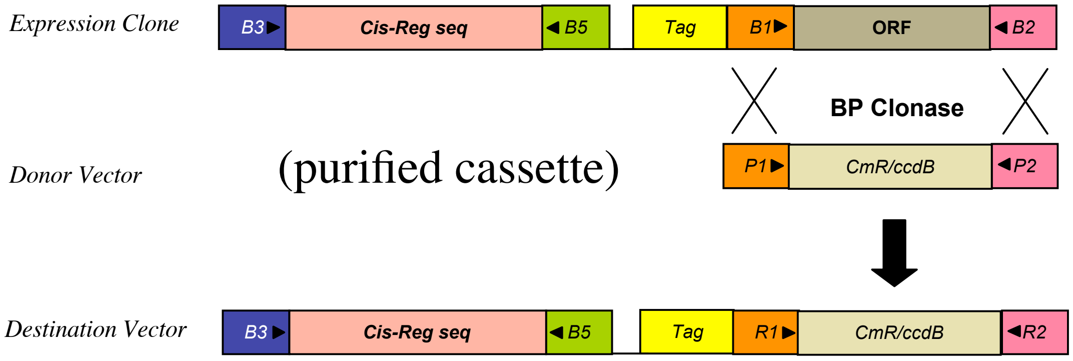

What you need:

1. A pSP72-B3-CisReg-B5::B1-ORF-B2 with or without tag (here an N-ter Tag is shown, but the vectors exist with N-ter, C-ter tag or without tag (see p19).
2. A purified pDONR-221-P3-P5 cassette to replace the Cis-reg sequence or a pDONR-221-P1-P2 purified cassette to replace the ORF of interest. Purification of the cassette is required for efficient retrieval of the correctly recombined pDest vector.
3. This results in pSP72-R3-ccdB/cmR-R5::B1-ORF-B2 or pSP72-B3-cisReg-B5::R1-A-R2 destination vectors.

Inserting a new cis-regulatory region or ORF in a Gateway destination vector.

Aim:
starting from a destination vector that already has an ORF or a cis-reg of interest inserted, add a new cis-reg sequence or a new ORF in the free R1-A-R2 or R3-ccdB/CmR-R5 cassette.
Selection of the expression clone is done on ampicillin in DH5α strain.

New Cis-reg sequence

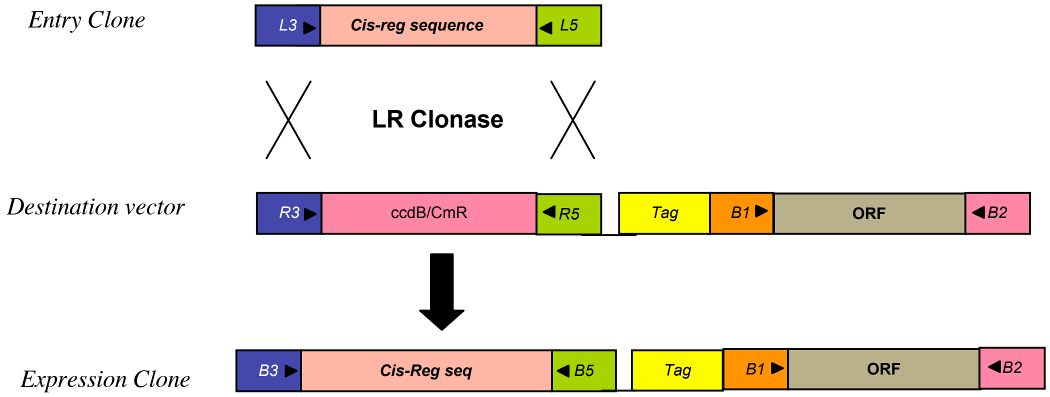

New ORF

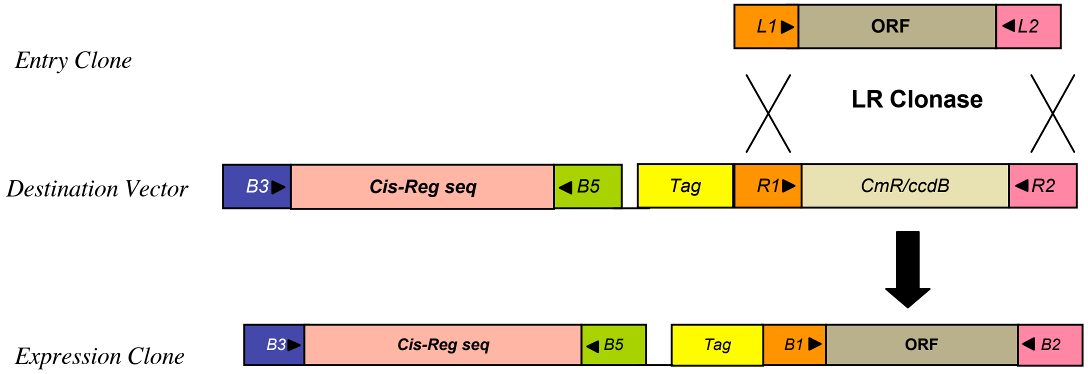

What you need:

1. A pSP72-R3-ccdB/cmR-R5::B1-ORF-B2 destination vector or a pSP72-B3-cisReg-B5::R1-A-R2 destination vector.
2. A pENTR-L3-New cisreg-L5 entry clone, or a pENTR-L1-NewORF-L2 entry clone with adequately designed sequences according to the tagging strategy used.

Cloning of an enhancer and minimal promoter in front of an ORF of interest.

<u>Aim:</u>
Starting from a destination vector that already has an ORF of interest inserted, recombine into the free R3-R5 cassette a complex cis-reg module, made of an enhancer and a minimal promoter.
Selection of the expression clone is on ampicillin in DH5α bacteria

<u>What you need:</u>

1. A pSP72-R3-ccdB/cmR-R5::B1-ORF-B2 destination vector.
2. A pENTR-L3-enhancer-L4 entry clone, and a pENTR-R4-MinProm-L5 entry clone with minimal or basal promoter.

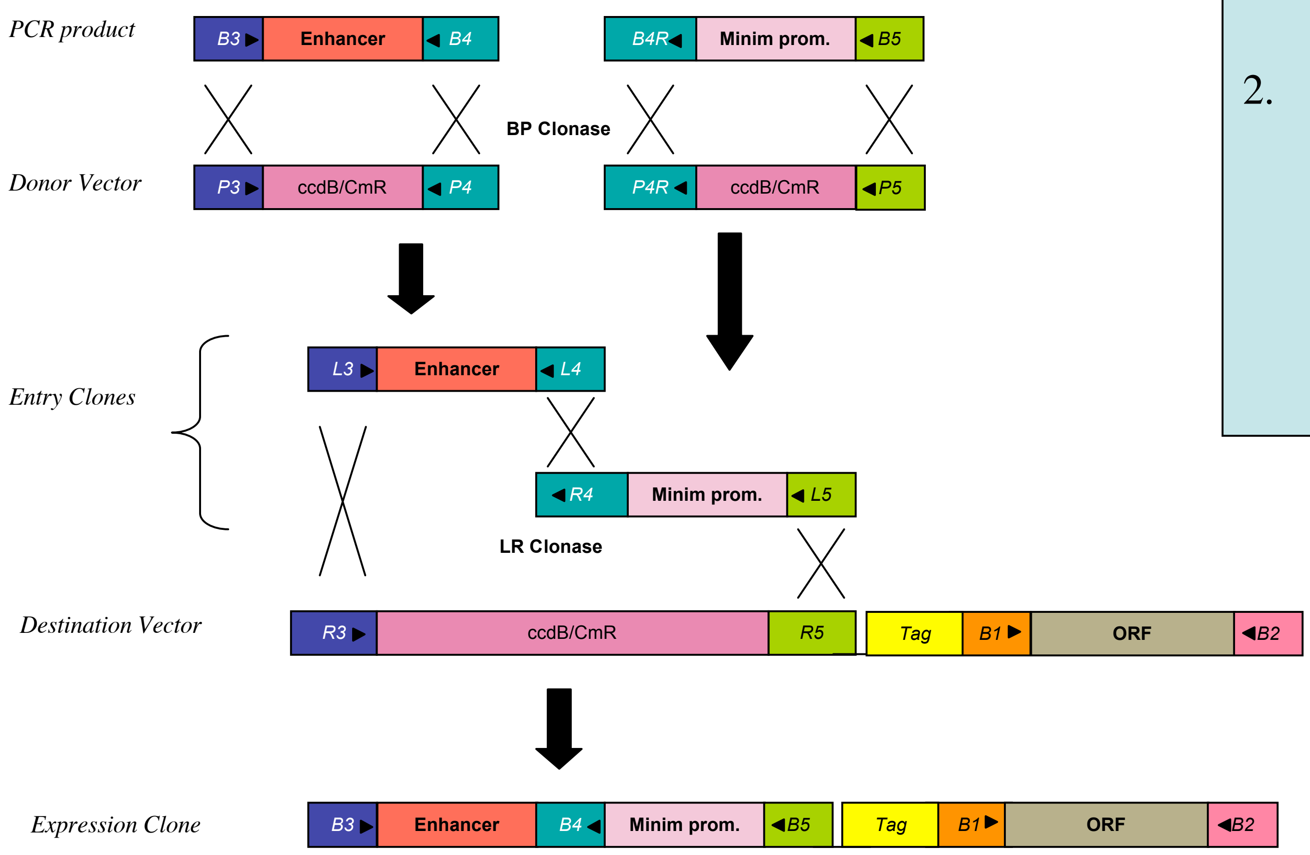

Position of distributed available vectors, Release May 2007

<u>Aim</u>: site position of each clone in the 96-well plate. For instance, « E6 » corresponds to pSPE3-RfA-HA in line E/column 6.

- ***Enrty clones to track cells and organelles :***

A1 -pENTR-L1-Kozak-GFP-GPI-stop-L2
B1 -pENTR-L1-Kozak-GAP43-GFP-stop-L2
C1 -pENTR-L1-Kozak-HistoneH2B-L2
D1 -pENTR-L1-Kozak-NLSlacZ-stop-L2
E1 -pENTR-L1-Kozak-LacZ-stop-L2
F1 -pENTR-L1-Kozak-Venus-stop-L2
G1 -pENTR-L1-Kozak-Ensconsin-3xGFP-stop-L2
H1 -pENTR-L1-Kozak-Ark-L2
A2 -pENTR-L1-Kozak-Dm-E-cadherin-L2

- ***Cis regulatory entry clones :***

B2 -pENTR-L3-pFOG-L5 (early ectodermal enhancer)
C2 -pENTR-L3-pSnail-L5 (mesodermal promoter)
D2 -pENTR-L3- Otx-a-element -L4 (early neural enhancer)
E2 -pENTR-L3- Titf -L4 (early endodermal enhancer)
F2 -pENTR-L3-eBra-L4 (notochord lineage enhancer)
G2 -pENTR-R4-bpFOG-L5 (strong basal promoter)

- ***Donor vector :***

A4 -pDONR-221-P1-P2
B4 -pDONR-221-P3-P5
C4 -pDONR-221-P3-P4
D4 -pDONR221-P4R-P5

- ***Destination vectors for mRNA synthesis :***

A6 -pSPE3-RfA
B6 -pSPE3-Venus-RfA
C6 -pSPE3-RfA-Venus
D6 -pSPE3-HA-RfA
E6 -pSPE3-RfA-HA

- ***Destination vectors for transgenesis (single cassette)***

F6 -pSP72BSSPE-Swa1::RfA
G6 -pSP72BSSPE-pFOGc::RfA
H6 -pSP72BSSPE-Swa1::Venus-RfA
A7 -pSP72BSSPE-Swa1::RfA-Venus
B7 -pSP72BSSPE-pFOGc::Venus-RfA
C7 -pSP72BSSPE-pFOG::RfA-Venus
D7 -pSP72BSSPE-Swa1::RfA-CFP
E7 -pSP72BSSPE-pFOG::RfA-CFP
F7 -pSP72BSSPE-Swa1::HA-RfA
G7 -pSP72BSSPE-Swa1::RfA-HA
H7 -pSP72BSSPE-pFOG::RfA-HA

- ***Destination vectors for transgenesis (2 cassettes)***

A9 -pSP72BSSPE-R3-ccdB/cmR-R5::RfA
B9 -pSP72BSSPE-R3-ccdB/cmR-R5::Venus-RfA
C9 -pSP72BSSPE-R3-ccdB/cmR-R5::RfA-Venus
D9 -pSP72BSSPE-R3-ccdB/cmR-R5::RfA-HA
E9 -pSP72BSSPE-R3-ccdB/cmR-R5::HA-RfA
F9 -pSP72BSSPE-R3-ccdB/cmR-R5::RfA-CFP
G9 -pSP72BSSPE-R3-ccdB/cmR-R5::RfA-Venus
H9 -pSP72BSSPE-R3-ccdB/cmR-R5::B1-NLSlacZ-B2
A10 -pSP72BSSPE-R3-ccdB/cmR-R4-bpFOGB5::B1-NLSLacZ-B2
