# Supplementary material for: A Multicassette Gateway Vector Set for High Throughput and Comparative Analyses in Ciona and Vertebrate Embryos
Source: PLoS One. 2007 Sep 19;2(9):e916. doi: 10.1371/journal.pone.0000916 (PMC1976267; doi:10.1371/journal.pone.0000916)
Supplement: Table S1 — (0.06 MB DOC) [file pone.0000916.s002.doc]

**TABLE S1 :  PRIMER SEQUENCES TO CONSTRUCT VECTORS**

| **Name** | **Primer sequence 5’to 3’** |
| --- | --- |
| pSPE3-Fw | AATTCAGGCCTTTGTTTAAACTTAGATATCGC |
| pSPE3-Rev | GGCCGCGATATCTAAGTTTAAACAAAGGCCTG |
| pSP72-Fw | AAATAGGCCTTTGTTTAAACTTAGAT |
| pSP72-Rev | ATCTAAGTTTAAACAAAGGCCTATTT |
| Apa1-Xba1-Fw | TCGGGCCCCTCTAGAAAATAATGA |
| attP3L Rev | CTCGTTCAACTTTATTATACAAAGTTGGCATTATAAAAAAG |
| attP3L Fw | CAACTTTGTATAATAAAGTTGAACGAGAAACGTAAAATG |
| Pst1 Rev | AGTCTGCAGGTCGATACAGTAGAAAT |
| EcoR1 Fw | CGGAATTCCGGATGAGCATTCATC |
| attP4L Rev | CAACTTTGTATAGAAAAGTTGAACGAGAAACGTAAAATG |
| attP5L Rev | CAACTTTGTATACAAAAGTTGAACGAGAAACGTAAAATG |
| EcoRV-Xba1- Rev | TAGGGGATATCATCTAGAGCTGGATGG |
| attP4L Fw | CTCGTTCAACTTTTCTATACAAAGTTGGCATTATAAGAAAG |
| attP5L Fw | GTTCAACTTTTGTATACAAAGTTGGCAGCATTATAAGAAAG |
| Apa1-attP4 Fw | GGGCCCTACAGGTCACTAATACCATC |
| Pst1-attP4 Rev | AACTGCAGTAGGGTCTAGAGATATCAGC |
