# Supplementary material for: A Multicassette Gateway Vector Set for High Throughput and Comparative Analyses in Ciona and Vertebrate Embryos
Source: PLoS One. 2007 Sep 19;2(9):e916. doi: 10.1371/journal.pone.0000916 (PMC1976267; doi:10.1371/journal.pone.0000916)
Supplement: Table S2 — (0.05 MB DOC) [file pone.0000916.s003.doc]

**TABLE S2 : PRIMERS SEQUENCES TO GENERATE TAGGED VECTORS**

| **Tag name** | **Position in vector** | **Sequences 5’ to 3’** | **Kozak** | **Stop codon** |
| --- | --- | --- | --- | --- |
| Venus | N-terminal | Fw : CAGAAAAAATGGTGAGCAAGGGCG  Rev : CTTGTACAGCTCGTCCATGCCG | Yes | No |
|  | C-terminal | Fw : ATGGTGAGCAAGGGCGAG  Rev : TTACTTGTACAGCTCGTCCAT | No | Yes |
| HA | N-terminal | Fw°: GACAAAAAATGGCATACCCATACGACGTGCCAGACTACGCT  Rev°: AGCGTAGTCTGGCACGTCGTATGGGTATGC CATTTTTTGTC | Yes | No |
|  | C-terminal | Fw°: TACCCATACGACGTGCCAGACTACGCTTGA  Rev°: TCAAGCGTAGTCTGGCACGTCGTATGGGTA | No | Yes |
| CFP | C-terminal | Fw : ATGGTGAGCAAGGGCGAG  Rev : TTACTTGTACAGCTCGTCCAT | No | Yes |

° these two couple of oligos were annealed and blunt subcloned in pSP72Swa ::RfA, EcoRV digested.
