# Supplementary material for: A Multicassette Gateway Vector Set for High Throughput and Comparative Analyses in Ciona and Vertebrate Embryos
Source: PLoS One. 2007 Sep 19;2(9):e916. doi: 10.1371/journal.pone.0000916 (PMC1976267; doi:10.1371/journal.pone.0000916)
Supplement: Table S3 — (0.09 MB DOC) [file pone.0000916.s004.doc]

**TABLE S3**- PRIMERS SEQUENCES USED TO GENERATE ENTRY CLONES

| **Name** | **From** | **Primer Sequence 5’ to 3’** | **Kozak** | **Stop**  **codon** |
| --- | --- | --- | --- | --- |
| GFP-GPI | Gift from A.Philips | GFP-GPI-attB1Fw**:**ggggacaagtttgtacaaaaaagcaggctCAGAAAAAATGGCCCTGTGGATGCGC  GFP-GPI-attB2Rev**:** ggggaccactttgtacaagaaagctgggtCTAAGTCAGCAAGCCCATG | Yes | Yes |
| GAP43-GFP | Miriyoshi and al., 1996 ; Kim and al., ,1998. | GAP43-GFP-attB1Fw : ggggacaagtttgtacaaaaaagcaggctCAGAAAAAATGCTGTGCTGTATGAGAAG  GAP43-GFP-attB2Rev : ggggaccactttgtacaagaaagctgggtTTACTTGTACAGCTCGTCCATG | Yes | Yes |
| LacZ | Corbo and al., 1997. | LacZ-attB1Fw: ggggacaagtttgtacaaaaaagcaggctCAGAAAAAATGCCGGTGGGTGAAGACCAG  LacZ-attB2Rev: ggggaccactttgtacaagaaagctgggtTTATTTTTGACACCAGACCAAC | Yes | Yes |
| NLSlacZ* | Corbo and al., 1997. | NLSlacZ-s-attB1Fw**:** aaaaagcaggctGCAAGGGTACCGAGCTCAG  NLSlacZ-s-attB2Rev**:** agaaagctgggtCAGACATGGCTTGCCCGG | Yes | Yes |
| Ensconsin-3XGFP | Lenart and al., 2005. | Ens-3GFP-attB1Fw :ggggacaagtttgtacaaaaaagcaggctCAGAAAAACTTATGGAGCAGAAGCTCATCTCAG  Ens-3GFP-attB2Rev : ggggaccactttgtacaagaaagctgggtTTACTTGTACAGCTCGTCCATGCCG | Yes | Yes |
| Dm-E-cadherin | Gift from JM Philippe.  Oda and al., 2001. | Dm-E-cad-attB1Fw: ggggacaagtttgtacaaaaaagcaggctCAACCATGTCCACCAGTGTCCAGCGA  Dm-E-cad-attB2Rev: ggggaccactttgtacaagaaagctgggtAGATGCGCCAGCCCTGGTCATC | Yes | No |
| Aurora Kinase * | This study | AurK-s-attB1Fw: aaaaagcaggctCAGAAAAAATGAGTTCTGCCAACAATGAAAACAAAGC  AurK-s-attB2Rev : agaaagctgggtTGTCCATAGGTCGACCATCTGGTC | Yes | No |
| Venus * | Gift from Miyawaki.  Nagai and al.,2002 | Venus-s-attB1Fw: aaaaagcaggctCAGAAAAAATGGTGAGCAAGGGCGAG  Venus-s-attB2Rev: agaaagctgggtTTACTTGTACAGCTCGTCC | Yes | Yes |
| Tau-lacZ | Gift from Hudson.  Montbaertz and al., 1987. | Tau-lacZ -attB1Fw: ggggacaagtttgtacaaaaaagcaggctCAGAAAAAATGGCTGAGCCCCGCCAG  Tau-lacZ-attB2Rev: ggggaccactttgtacaagaaagctgggtTTATTTTTGACACCAGACCAAC | Yes | Yes |
| Histone2B | Koster and al., 2001. | H2B-attB1Fw: ggggacaagtttgtacaaaaaagcaggctCAGAAAAAATGCCAGAGCCAGCGAAG  H2B-attB2Rev: ggggaccactttgtacaagaaagctgggtTCTTAGCGCTGGTGTA CTTG | Yes | No |
| GATAa  (fusion with C-ter tag) | Bertrand and al., 2003. | NGATA-attB1Fw: ggggacaagtttgtacaaaaaagcaggctCAGAAAAAATGTATATGCCTAACCCCG  NGATA-attB2Rev: ggggaccactttgtacaagaaagctgggtCTATCACTTCAGGTTTAACATA | Yes | No |
| GATAa  (fusion with N-ter tag) | Bertrand and al., 2003. | CGATA-attB1Fw:ggggacaagtttgtacaaaaaagcaggctTGATGTATATGCCTAACCCCG  CGATA-attB2Rev: ggggaccactttgtacaagaaagctgggtTTATATCACTTCAGGTTTAAC | No | Yes |
| « a- element » *  (-1541 to -1418 of Otx cis-regulatory region) | Bertrand and al., 2003. | a- element-s-attB3Fw: ataaagtaggctCGTTATCTCTAACGGAAGTTTTC  a- element-s-attB4Rev: gaaaagttgggtTCCTATCTTAGATATTGAAC | No | No |
| Enhancer Brachury *  (-470 to -61 of Brachury cis-regulatory region) | Yagi and al., 2004. | eBra-s-attB3Fw : ataaagtaggctAACACACCCAACGTACAATAAAAC  eBra-s-attB4Rev : gaaaagttgggtCTTCTTTTTGAAATTTTATGTTTG | No | No |
| bpFOG  (-349 to -1 of FOG cis regulatory region) | Rothbacher and al., in press | bpFOG-attB4RFw: ggggccaagttttctatacaaagtggcaAAGCTTCGTGTATTGTACGG  bpFOG-attB5Rev: ggggaccactttgtatacaaaagttgggtTATGTGTGTTATTTTTGTATAG | No | No |
| pFOG  (-1981 to -1 of FOG cis-regulatory region) | Rothbacher and al., in press | FOG-attB3Fw :ggggacaagtttgtataataaagtaggctGCAACTATTGTAACACCACAC  FOG-attB5Rev : ggggaccactttgtatacaaaagttgggtTATGTGTGTTATTTTTGTATAGAC | No | No |
| pSna *# | Erives and al., 1998. | pSna-s-attB3Fw : ataaagtaggctCATGATAAATTAATATGTTGTTACCGATA  pSna-s-attB5Rev caaaagttgggtGCATGGGCTCGACGGAGGTC | No | No |
| pTitf1 *$ | Fanelli and al., 2003. | pTitf1-s-attB3Fw :ataaagtaggctCACCGCTCGATGGGGCTCAAG  pTitf1-s-attB4Rev : gaaaagttgggtATTTTAACCCTGCAGGCCAAACTTG | No | No |

* : Entry clones which were generated by nested PCR: the first PCR was performed with « s-attB » primer and the second with the corresponding « attB-adaptor » primers (Suppl. Mat 1, page 16).

# 881bp fragment extending from 795 bp upstream to 86 bp dowstream of the 5’ end of Ci-snail mRNA (genbank ID : AF002987)

$ : 949 bp fragment, extending in its 5’ end from +2210 to 2907 of genomic Titf region (genbank ID : AJ515710), and including in its 3’ end a 257 bp repetitive element present in the French ascidian population at this locus.
